# Tumor Necrosis Factor-Like Weak Inducer of Apoptosis (TWEAK) Enhances Activation of STAT3/NLRC4 Inflammasome Signaling Axis through PKCδ in Astrocytes: Implications for Parkinson’s Disease

**DOI:** 10.3390/cells9081831

**Published:** 2020-08-04

**Authors:** Manikandan Samidurai, Prashant Tarale, Chelva Janarthanam, Crystal Gomez Estrada, Richard Gordon, Gary Zenitsky, Huajun Jin, Vellareddy Anantharam, Anumantha G. Kanthasamy, Arthi Kanthasamy

**Affiliations:** 1 Department of Biomedical Sciences, Iowa Center for Advanced Neurotoxicology, Iowa State University, Ames, IA 50011, USA; manisk@iastate.edu (M.S.); ptarale@iastate.edu (P.T.); chelvaj@iastate.edu (C.J.); crystalgomezestrada@gmail.com (C.G.E.); zenitsky@iastate.edu (G.Z.); egb761@iastate.edu (H.J.); anantram@iastate.edu (V.A.); akanthas@iastate.edu (A.G.K.); 2 School of Biomedical Sciences, University of Queensland, St Lucia, Queensland 4072, Australia; r.gordon1@uq.edu.au

**Keywords:** TWEAK, STAT3, NLRC4, PKCδ, astrocyte, mitochondrial dysfunction, Parkinson’s disease

## Abstract

Astrocytic dysfunction has been implicated in Parkinson’s disease (PD) pathogenesis. While the Tumor necrosis factor-like weak inducer of apoptosis (TWEAK)/Fn14 signaling axis is known to play a role in PD-like neuropathology, the molecular mechanisms that govern this process remain poorly understood. Herein, we show that TWEAK levels are elevated in PD serum compared to controls. Moreover, using both U373 human astrocyte cells and primary mouse astrocytes, we demonstrate that TWEAK induces mitochondrial oxidative stress as well as protein kinase C delta (PKCδ) and signal transducer and activator of transcription 3 (STAT3) activation, accompanied by NLRC4 inflammasome activation and upregulation and release of proinflammatory cytokines, including IL-1β, TNF-α, and IL-18. Mechanistically, TWEAK-induced PKCδ activation enhances the STAT3/NLRC4 signaling pathway and other proinflammatory mediators through a mitochondrial oxidative stress-dependent mechanism. We further show that PKCδ knockdown and mito-apocynin, a mitochondrial antioxidant, suppress TWEAK-induced proinflammatory NLRC4/STAT3 signaling and cellular oxidative stress response. Notably, we validated our in vitro findings in an MPTP mouse model of PD and in mice receiving intrastriatal administration of TWEAK. These results indicate that TWEAK is a key regulator of astroglial reactivity and illustrate a novel mechanism by which mitochondrial oxidative stress may influence dopaminergic neuronal survival in PD.

## 1. Introduction

Parkinson’s disease (PD) is an age-related neurodegenerative disorder that profoundly impacts nigrostriatal dopamin(DA)ergic neurons [1,2]. The clinical manifestations of PD include resting tremors, bradykinesia and muscular rigidity. The nonmotor symptoms of PD such as sleep disorders, depression and cognitive changes are known to occur during the early phase of the disease and have been used for early diagnosis [3]. The precise cause of Parkinson’s disease (PD) remains unknown; however, recent literature points to mitochondrial dysfunction, chronic inflammation and oxidative stress as the pivotal mechanisms of PD neuropathology [4]. Additionally, environmental risk factors such as carbon disulfide, cyanide, herbicides, methanol/organic solvents, pesticides and manganese have been implicated in PD-associated DAergic neuronal cell death [3,5].

Accumulating evidence supports a role for a chronic inflammatory response in the progressive loss of DAergic neurons in animal models of PD [6,7]. Epidemiological and genetic analyses have revealed an association between neuroinflammation and PD pathophysiology [8]. In fact, activated microglial cells, T-lymphocytes and proinflammatory mediators have been identified in the substantia nigra (SN) of PD patients. Other characteristic features of inflammation such as complement activation, phagocyte activation and increased synthesis and release of proinflammatory cytokines have also been identified in cellular and animal models of PD [9]. In this context, specific proinflammatory factors such as tumor necrosis factor-alpha (TNF-α) and IL-1β are upregulated in different brain regions of PD patients [10]. These proinflammatory processes in PD patients are considered to be instrumental in promoting the progression of deleterious events that facilitate DAergic neuronal loss [11]. 

Astrocytes are recognized as the most abundant cell type in the CNS and function to provide metabolites and growth factors, participate in synapse formation, support plasticity, and maintain extracellular ion balance and neurotransmitter levels [12]. Their critical contribution to the maintenance of the permeability of the blood-brain barrier (BBB) and their active role in the removal of extracellular glutamate makes them key regulators of immune cell trafficking and its subsequent activation. Astrocytes are immune-competent cells capable of danger-signal detection through the secretion of chemokines and cytokines, which activate the innate immune response [13]. Their role in neuroinflammation has been dissected using transgenic mice that carry mutations in astrocyte proteins involving transcription, cell communication and ligand binding. These models have identified signals that trigger damaging pathways within astrocytes such as IL-17, sphingolipids, TrkB, SOCS3, NFĸB, chemokines and VEGF in astrocytosis [14]. In fact, age-related inflammation has been associated with the chronic activation of astrocytes [15]. To date, despite a growing literature supporting a positive association between PD neuropathology and astrocyte activation, the exact role of astrocyte-derived proinflammatory factors in eliciting innate immunity remains enigmatic. 

A plethora of signaling pathways are known to induce the expression of proinflammatory genes [16]; however, phosphorylation of STAT3 has been reported to be critical for inducing expression of glial fibrillary acidic protein (GFAP) as well as proinflammatory genes including TNF-α and IL-6 [17,18,19]. Thus, STAT3 may play an important role in the activation of astrocytes and associated inflammatory pathways. Inflammasomes are multiprotein complexes that are assembled in the cytosol in response to pathogen-associated molecular patterns (PAMPs) or damage-associated molecular patterns (DAMPs). Several inflammasome-forming, pattern recognition receptors (PRRs) have been recognized, including: NLR family, pyrin domain-containing 1 (NLRP1); NLRP3; NLRP6; NLRP12; NLR family, CARD domain-containing 4 (NLRC4); AIM2 (absent in melanoma 2); and RIG-1 [20]. Upon recognizing PAMPs or DAMPS, NLR or AIM2 oligomerize, leading to the autocatalytic activation of pro-caspase-1 proteins, resulting in the secretion of mature IL-1β and IL-18 [21]. To date, NLRP3 inflammasome activation is the most studied inflammasome in neurodegenerative diseases, including PD. Accordingly, NLRP3 knockout (KO) mice were resistant to 1-methyl4-phenyl-1,2,3,6-tetrahydropyridine (MPTP)-induced caspase-1 activation, IL-1β generation and IL-18 release [22]. Likewise, we and others have demonstrated NLRP3 inflammasome activation in postmortem PD brains and mouse models of PD [23,24,25]. Additionally, previous studies using mouse models and human data have demonstrated the pivotal role of the inflammasome in the progression and development of neurodegenerative diseases such as AD [26,27]. Notably, the NLRC4 inflammasome has been shown to elevate neuronal Aβ accumulation through proinflammatory astrocytic activation [28]. Proinflammatory stimuli, such as TNF-α, are also known to upregulate the expression of the NLRC4 inflammasome [29]. Despite these studies, the mechanisms underlying NLRC4 inflammasome activation in astrocytes and its relationship to PD-like pathology remain poorly characterized. 

TNF-like weak inducer of apoptosis (TWEAK) and its cognate receptor, fibroblast growth factor inducible 14 (Fn14), belong to the TNF receptor superfamily [30,31]. TWEAK-Fn14 signaling has been posited to regulate different cell functions, including cell proliferation, migration and survival in a context-specific manner [32]. Relatively low levels of expression of both TWEAK and its receptor Fn14 are evidenced under normal conditions [33]; however, its levels are upregulated under pathological conditions including in a neonatal mouse model of hypoxic ischemia [31]. Additionally, TWEAK and its receptor Fn14 have been implicated in other pathological conditions, including ischemic stroke and multiple sclerosis [33,34,35,36]. Interestingly, a recent study demonstrated the involvement of the TWEAK-Fn14 axis in DAergic neurodegeneration in an MPTP mouse model of PD [37], pointing to TWEAK-Fn14 as a potential contributor to neurodegeneration. Notably, a previous study demonstrated the proinflammatory role of TWEAK in cultured astrocytes in culture [38]. Moreover, TWEAK promotes astrocyte proliferation with an accompanying enhancement of experimental autoimmune encephalomyelitis (EAE) severity in mice overexpressing soluble TWEAK [36]. Owing to this evidence, in the present study, we tested the effect of TWEAK on astroglial reactivity and its relationship to DAergic neurodegeneration in the context of a preclinical experimental model of PD. 

PKCδ is involved in cell cycle progression, apoptosis, and cell proliferation and differentiation [39]. Previously, we demonstrated that PKCδ is highly expressed within the DAergic neurons in the SN [40,41]. Proteolytic activation of PKCδ mediated by caspase-3 has been found to cause apoptosis upon stimulation of DAergic neurons with TNF-α [40]. In contrast, PKCδ was found to be activated via enhancement of its kinase activity upon stimulation of microglial cells with diverse inflammatory stimuli, suggesting cell type-specific effects with regards to the PKCδ activation mechanism [39]. Importantly, genetic ablation of PKCδ results in the attenuation of both nitric oxide production and cytokine/chemokine release in microglial cells exposed to an inflammatory stimulus, suggesting that PKCδ is a critical determinant of the microglial activation response [39,42]. However, the exact contribution of PKCδ in the astrocytic activation response remains elusive. In this study, we examined the cell signaling events underlying the TWEAK-induced astroglial activation state and its relationship to PD-like pathology. In this context, we characterized the relevant PKCδ-mediated proinflammatory signaling events and mitochondria-dependent oxidative stress mechanisms that contribute to an aberrant astroglial activation state in response to TWEAK treatment. Our in vitro findings demonstrate that downregulation of PKCδ and pharmacological inhibition of mitochondrial oxidative stress ameliorated the TWEAK-induced astroglial activation state by suppressing NLRC4 inflammasome and STAT3 activation. Intriguingly, mito-apocynin reduced DAergic neurotoxicity mediated by TWEAK-stimulated astrocytes. Importantly, increased expression of TWEAK within reactive astrocytes positively correlated with PKCδ and NLRC4 inflammasome activation as well as pronounced TH neuronal loss in the SN of MPTP-treated mice. Thus, therapeutic interventions targeted against PKCδ and mitochondria-dependent oxidative stress mechanisms may represent novel disease-modifying strategies to halt or slow the progression of PD.

## 2. Methods and Materials

### 2.1. Chemicals and Biological Reagents

Recombinant TWEAK was obtained from PeproTech. Modified Eagle’s medium (MEM), fetal bovine serum (FBS), L-glutamine, MitoSox, DCFDA-H2 kit for ROS assay, IRDye-tagged secondary antibodies, Hoechst nuclear stain, penicillin, streptomycin and other cell culture reagents were purchased from Invitrogen (Carlsbad, CA, USA). ELISA kits for IL-1β, IL-18, TNF-α and TWEAK were purchased from RayBiotech (Peachtree Corners, GA, USA). Rabbit anti-NLRC4, PKCδ, pPKCδ-Y311, STAT3 and pSTAT3 antibodies were purchased from Cell Signaling Technology (Danvers, MA, USA). ASC and caspase-1 antibodies were purchased from AdipoGen (San Diego, CA, USA). GFAP antibody was purchased from EMD Millipore (Burlington, MA, USA). Aurintricarboxylic acid (ATA), 1-methyl-4-phenylpyridinium (MPP^+^ iodide), MPTP-HCl, and β-actin antibody were purchased from Sigma-Aldrich (St. Louis, MO, USA). Mito-apocynin was obtained from Dr. Kalyanaraman (Medical College of Wisconsin, Milwaukee, WI, USA). MitoTracker Red CMXRos was purchased from Molecular Probes (Eugene, OR, USA). Luminex multiplex cytokine (Multiplex mouse cytokine panel) assay kit was purchased from Bio-Rad Laboratories (Hercules, CA, USA). The CD11b magnetic separation kit was purchased from Stem Cell Technologies (Vancouver, Canada). The Bradford protein assay and TidyBlot detection reagents were purchased from Bio-Rad Laboratories.

### 2.2. Human Astrocyte (U373) Cell Culture and Treatment

The human astrocytic U373 cell line was obtained from ATCC and maintained in MEM medium supplemented with 10% heat-inactivated FBS, 50 U/mL penicillin, 50 μg/mL streptomycin, 2 mM L-glutamine and 2 mM sodium pyruvate (Invitrogen) at 37 °C with 5% CO_2_ humidity. U373 cells were treated with TWEAK (100 ng/mL) for the indicated time (6, 12, 18, 24 h), mito-apocynin at 10 µM for 1 h and ATA at 10 µM for 1 h. Treatments for the U373 astrocytic cell line were performed in 2% FBS-containing MEM. At the end of the incubation period, cells were subjected to various experiments as detailed below, and their media were collected for the measurement of cytokines.

### 2.3. LUHMES Human Dopaminergic Neuronal Cell Culture and Treatment

For this study, the Lund human mesencephalic (LUHMES) cell line derived from female human embryonic ventral mesencephalic cells was obtained from the American Type Culture Collection. LUHMES cells were grown as described previously [43]. Undifferentiated LUHMES cells were grown in Advanced DMEM/F-12 supplemented with 1× N-2 supplement, 2 mM l-glutamine and 40 ng/mL recombinant basic FGF on plastic flasks or multi-well plates precoated with 50 μg/mL poly-l-ornithine and 1 μg/mL fibronectin at 37 °C with 5% CO_2_ humidity. LUHMES cells were treated with the astrocyte-conditioned media (ACM) for another 12 h. ACM obtained from the U373 cells treated with TWEAK in the presence or absence of mito-apocynin were collected and applied to LUHMES cells for assessment of cell viability. At the end of the incubation period, cells were subjected to MTS cell viability assay.

### 2.4. Primary Astrocyte Culture

Primary astrocyte cultures were prepared from wild-type (WT) postnatal day 1 mouse pups based on a technique described in a previous publication with slight modifications [44]. Brains harvested from mice had their meninges removed before being placed in DMEM/F12 supplemented with 10% heat-inactivated FBS, 50 U/mL penicillin, 50 µg/mL streptomycin, 2 mM L-glutamine, 100 µM nonessential amino acids and 2 mM sodium pyruvate. Brain tissues underwent gentle agitation in 0.25% trypsin-EDTA for 15 min. Double the volume of DMEM/F12 complete medium was used to stop trypsin activity, followed by washing the brain tissues three times. Tissues were then triturated gently to prepare a single-cell suspension, which was passed through a 70-µm nylon mesh cell strainer to remove debris. The cell suspension was then made up in DMEM/F12 complete medium and seeded into T-75 flasks, which were incubated in humidified 5% CO_2_ at 37 °C. As described previously [44], astrocyte cells were separated from confluent mixed glial cultures by CD11b magnetic-bead separation to 97% purity and were then allowed to recover for 48 h after plating. Upon isolation of microglia using the magnetic bead technology, the negative fraction, which was predominantly astrocytes, was plated in T-75 flasks containing the growth medium. After growing the cells overnight in the growth medium in a 37 °C incubator, astrocytes were split the following day for different experimental treatment conditions.

### 2.5. Animal Treatment

Six- to eight-week-old C57 black mice were obtained from Charles River and housed under standard conditions at 22 ± 1 °C, 30% relative humidity and a 12-h light cycle as per Institutional Animal Care and Use Committee (IACUC) protocol. Mice were randomly assigned into two different groups (Control, TWEAK). An acute intrastriatal TWEAK-induced neuroinflammation model was used for this study [45]. Mice were given intrastriatal injections of 2 µL TWEAK (1 µg/µL) using stereotaxic surgery. Mice were sacrificed 24 h posttreatment to collect the striatum and SN, which were stored at −80 °C until tissue lysates could be prepared for immunoblotting the protein of interest. The use of animals and all animal-related procedures in this study (Protocol ID IACUC-19-259) were approved and supervised by the IACUC at Iowa State University, Ames, IA, USA.

### 2.6. MPTP Mouse Model

Male C57BL/6 mice, aged 10–12 weeks and weighing 24–28 g, were housed under standard conditions as described above. Mice were intraperitoneally administered 25 mg/kg of MPTP daily for 5 days. Control animals received equal volumes of sterile saline solution. All groups were sacrificed at the end of 7 days after the last dose of either saline or MPTP. Then, the brains were harvested for immunohistochemistry (IHC) or Western blotting (WB) analysis.

### 2.7. Human Sample Analysis

Serum samples from patients with PD and age-matched controls were obtained from the University of California (Los Angeles, USA). The average age of PD patients was 80 ± 7.65 years, and the unaffected controls averaged 80 ± 7.85 years. The samples were analyzed by an investigator blinded to the experimental conditions. Serum TWEAK concentrations were determined using an ELISA kit according to the manufacturer’s instructions. Briefly, the 100 µL samples were incubated in 96-well plates coated with primary antibodies for 3 h at room temperature (RT). After incubation, the samples were washed and incubated with detection antibody and biotin/streptavidin. Plates were read at 450 nm immediately followed by adding the STOP solution. For WB analysis, the serum samples were diluted and passed through a column (Pierce, Waltham, MA, USA) to remove the IgG and albumin first, then concentrated by Pierce Protein Concentrators (Thermo Scientific, Waltham, MA, USA). Equal (60 µg) amounts of protein were loaded for each sample and separated using a 12% SDS-PAGE gel for 2 h at 110 V. Upon completion of the separation, the proteins were transferred to a nitrocellulose membrane and the nonspecific-binding sites were blocked using 5% BSA for 1 h at RT. Membranes were then probed overnight at 4 °C with the respective primary antibodies. TidyBlot detection reagent was used to avoid the visualization of denatured IgG.

### 2.8. Immunohistochemistry and Immunofluorescence

IHC was performed using sections from the SN and other brain regions of interest as described previously [46,47]. A mixture of 200 mg/kg ketamine and 20 mg/kg xylazine was used to deeply anesthetize the mice for transcardial perfusion with freshly prepared 4% paraformaldehyde (PFA) solution. Extracted brains were post-fixed in 4% PFA for 48 h. Next, 30-µm sections were cut using a freezing microtome (Leica Microsystems, Wetzlar, Germany) and 5-µm paraffin-embedded sections were cut on a normal microtome by the Department of Pathology, College of Veterinary Medicine, ISU. Sections underwent antigen retrieval using citrate buffer (10 mm sodium citrate, pH 8.5) for 30 min at 90 °C. Sections were blocked with PBS containing 2% BSA, 0.2% Triton X-100 and 0.05% Tween 20 for 1 h at RT. Sections were probed with primary antibodies for GFAP (1:1000, mouse monoclonal) and TWEAK (1:500, goat polyclonal; Santa Cruz) overnight at 4 °C. The next day, they were washed 7 times for 5 min each in PBS on a Belly Dancer shaker (SPI Supplies, West Chester, PA, USA). Appropriate Alexa Fluor-conjugated secondary antibodies (Alexa Fluor 488 or 594 or 555) were used to probe the sections for 60 min at RT, and their cell nuclei were stained with a Hoechst dye. Sections were mounted on slides using Prolong antifade gold-mounting medium (Invitrogen) according to the manufacturer’s instructions. 

Coronal SN sections were performed with DAB immunostaining, as described previously with slight modifications [46]. Briefly, 30-μm sections were incubated with an anti-TH antibody (1:1000, mouse monoclonal; EMD Millipore), followed by incubation with the respective biotinylated secondary antibody. Total numbers of TH^+^ neurons in every sixth section of the SN were counted stereologically with the Stereo Investigator software (MBF Bioscience, Williston, VT, USA) [48] and then averaged over the total number of sections per animal [49]. 

Immunofluorescence studies in U373 cells were performed based on previously published protocols with few modifications [50]. Approximately 25,000 cells were plated onto Poly-d-Lysine (PDL)-coated coverslips. After treatments, cells were fixed with 4% PFA, washed in PBS and blocked with buffer (PBS containing 2% BSA, 0.5% Triton X-100, and 0.05% Tween-20) for 1 h at RT. Coverslips containing cells were probed with the respective primary antibodies GFAP (1:1000, mouse monoclonal) and STAT3 (1:500, goat polyclonal; Santa Cruz) diluted in PBS containing 1% BSA and incubated overnight at 4 °C. Coverslipped-cells were washed several times in PBS and incubated with Alexa Fluor 488 and Alexa Fluor 555 dye-conjugated secondary antibodies. The nuclei were counterstained using Hoechst stain (10 µg/mL), and coverslips were mounted with Fluoromount medium (Sigma-Aldrich) on glass slides for visualization. Samples were visualized using an inverted fluorescence microscope (Nikon, Tokyo, Japan), and images were captured using Keyence BZ-0023.

### 2.9. Live Cell Staining

After the respective treatments, cells were stained using MitoSox Red (Molecular Probes) following the manufacturer’s protocol and a process described in our previous publication [51]. Briefly, cells were plated on PDL-coated coverslips or in 96-well plates. Following treatment, cells were washed twice with HBSS, and then the MitoSox dye was added according to the manufacturer’s recommended dilution. After staining, cells were washed and then either fixed for ICC or imaged directly. For MitoSox Red, the Keyence imaging system was used to take images after the treatment. 

### 2.10. Mitochondrial Fragmentation Analysis

U373 cells were grown on glass coverslips coated with 0.1% PDL. After treatment, the cells were incubated with the MitoTracker Red dye at a dilution of 1:7000 from a stock of 1 mM dye for 12 min at 37 °C. After incubation, cells were fixed with 4% PFA for 1 h and then washed several times with PBS. The Keyence imaging system was used to take images after the treatment. Quantification of mitochondrial morphology was conducted using the ImageJ plugin “mitochondrial morphology,” developed by Ruben K Dagda. We selected parameters for quantifying damage to mitochondrial structural features based on recent studies [51,52].

### 2.11. Multiplex Cytokine Luminex Immunoassays

Multiple cytokine assays were performed according to our previous publication [23] with some modifications. After treatment, mice were sacrificed, and the brain regions of interest were harvested and stored at −80 °C. The tissue lysates were subsequently subjected to tissue homogenization. Briefly, 50 µL samples were incubated with multiplex conjugated with primary antibodies for 1 h at RT. After the incubation, the samples were washed and incubated with the detection antibody and biotin/streptavidin. A Bio-Plex plate reader was used to assay and acquire the data. 

### 2.12. Intracellular Reactive Oxygen Species (iROS) and MitoSox Assays

U373 astrocyte cells (20,000 cells per well) were seeded in a 96-well plate and treated with TWEAK (100 ng/mL) at various time points after cell attachment. The media was then removed, and 1 µM of redox-sensitive CM-H2DCFDA dye in HBSS containing 2% FBS was added and incubated for 30 min at 37 °C. The supernatant with unabsorbed dye was removed, and cells were washed by adding PBS. Fluorescence was measured using excitation and emission wavelengths of 488 and 525 nm, respectively. The fluorescence value from the control cultures was subtracted as background, and the increase in fluorescence with treatments served as the measurement of increased iROS. The MitoSox assay was conducted by incubating the posttreatment cells with 5 µM MitoSox dye for 20 min and washing them twice with HBSS buffer. Results were read by using a fluorescence microplate reader with excitation and emission wavelengths of 510 and 580 nm, respectively.

### 2.13. Astroglial Nitric Oxide Detection

Nitric oxide production by U373 cells was measured indirectly by quantifying nitrite in the supernatant using the Griess reagent (Sigma-Aldrich). Then, 24 h post-TWEAK treatment, 100 μL of supernatant from each treatment was mixed with an equal volume of Griess reagent in a separate 96-well plate. The samples were then incubated on a plate shaker at RT for 15 min until their color stabilized. The absorbance was measured at 540 nm using a Synergy 2 multimode microplate reader (BioTek Instruments, Winooski, VT, USA). The nitrite concentration was determined relative to a sodium nitrite standard curve.

### 2.14. MTS Assay

MTS assay was performed according to the method described in our previous publications [51,53]. Briefly, 20,000 cells/well were seeded in a 96-well tissue culture plate and exposed to 100 µL of treatment medium after attachment. Cells were then processed using the Cell Titer 96^®^ Aqueous One Solution Cell Proliferation (MTS) Assay kit. After a 45-min (37 °C) incubation with 10 μL MTS dye, the formazan crystals were dissolved with 25 μL of dimethyl sulfoxide (Sigma-Aldrich), and the change in color was measured spectrophotometrically at 490 nm; values were bar graphed as % of control.

### 2.15. ELISA

U373 astrocytes (1 × 10^5^ cells/well) were plated in 96-well plates. Cells were incubated for 24 h for attachment. The next day, cells were treated with TWEAK (100 ng/mL), mito-apocynin (10 µM) or ATA (10 µM). At the end of the treatment period, the supernatant was collected and assessed for secreted cytokines, including IL-1β, IL-18 and TNF-α using the ELISA kit according to the manufacturer’s instructions. Briefly, the 100 µL samples were incubated in a 96-well plate coated with primary antibodies for 3 h at RT. After incubation, the samples were washed and incubated with the detection antibody and biotin/streptavidin. The plate was read at 450 nm immediately, followed by adding the STOP solution.

### 2.16. Real-Time Quantitative Reverse Transcription PCR (qRT-PCR)

Cells were harvested at the end of the treatment period, and RNA was extracted using TRIZOL reagent (Invitrogen, Carlsbad, CA, USA) as per the manufacturer’s protocol. RNA was quantified with an ND1000 NanoDrop spectrophotometer (Thermofisher Scientific, Waltham, MA, USA). A High-Capacity cDNA synthesis kit (Applied Biosystems, Waltham, MA, USA) was used to convert 600 ng of RNA to cDNA. Real-time qPCR was carried out on Applied Biosystems QuantStudio 3 with SYBR master mix (Invitrogen, Carlsbad, CA, USA). QuantiTect Primer Assays (Qiagen, Germantown, MD, USA) for genes specific to the proinflammatory cytokines TNF-α, IL-1β and IL-18 were used for qRT-PCR. The housekeeping gene 18S rRNA (Qiagen#PPM57735E) was used as the reference for all qRT-PCR experiments. Dissociation curves were run to make sure a single amplicon peak was obtained for respective genes primers. Fold change was calculated using the 2-∆∆Ct method.

### 2.17. Western Blotting 

These studies were carried out according to a previously published procedure from our lab [39]. Astrocytic cell lysates were prepared using modified RIPA buffer and normalized for equal amounts of protein loading using a Bradford protein assay. Equal amounts of protein (30 µg) were loaded for each sample and separated using a 12% SDS-PAGE gel. Upon completion of the separation, the proteins were transferred to a nitrocellulose membrane and the nonspecific-binding sites were blocked using a blocking buffer. Membranes were then probed overnight at 4 °C with the respective primary antibodies pPKCδ, PKCδ, STAT3, pSTAT3, NLRC4 (1:1000, Rabbit monoclonal), GFAP (1:1000, mouse monoclonal) and ASC (1:1000, Rabbit polyclonal; EMD Millipore), Caspase-1 (1:1000, mouse polyclonal). Following incubation, the membranes were washed 3 times with PBS, and then the membranes were visualized using IRDye-tagged secondary antibodies on the Odyssey infrared imaging system (LI-COR); β-actin (1:10000, mouse monoclonal) was used as a loading control.

### 2.18. siRNA Transfection

The predesigned PKCδ siRNA was purchased from Santa Cruz. U373 cells were used in the siRNA transfections with Lipofectamine 3000 reagent according to the manufacturer’s protocol. U373 cells were plated at 2 × 10^6^ cells/well in 6-well plates one day before transfection. For each well, 700 pM of PKCδ siRNA or an equal amount of scrambled siRNA mixed with 5 µL of Lipofectamine 3000 was added to the cells. Then, 48 h after the initial transfection, cells were treated with TWEAK for 24 h. Immunoblots were performed to check for successful transfections.

## 3. Data Analysis

Results were analyzed using two-tailed *t*-tests, i.e., either one-way or two-way ANOVA followed by Bonferroni’s post hoc analysis (PRISM 6.0 software, GraphPad, La Jolla, CA, USA), and are represented as the mean ± SEM. Differences were considered statistically significant for *p*-values ≤ 0.05.

## 4. Results

### 4.1. TWEAK Expression is Elevated in the Serum of Human PD Patients

**Figure 1:**
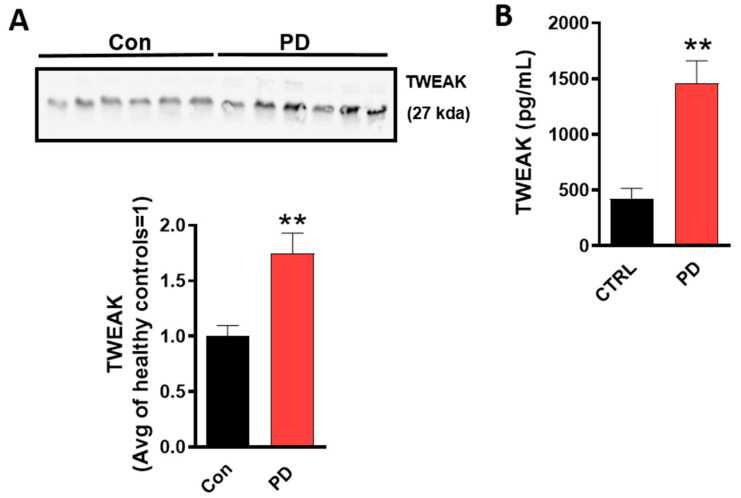
TWEAK expression is elevated in serum from PD patients. Representative immunoblots for TWEAK in serum from PD and control subjects. (**A**) Densitometric scanning analysis demonstrates elevated TWEAK levels in PD serum as compared with control subjects. The band intensity of TWEAK serum concentration corresponding to PD patients has now been normalized to the average intensity of healthy control subjects (non-PD). Data shown are the mean ± SEM from at least ten individual patients’ samples. (**B**) Confirmation of elevated TWEAK levels in PD serum samples as compared to controls using commercially available ELISA kit. Data shown are the mean ± SEM from at least ten individual patients’ samples. Data were analyzed using two-tailed *t*-test. Asterisks (** *p* < 0.01) indicate significant differences between control and treatment groups.

Serum levels of TNF-α and IL-1β were previously reported to be higher in PD patients than in controls [54,55]; this prompted us to investigate whether TWEAK would be elevated in the serum of PD patients. We, therefore, determined the expression levels of TWEAK in serum from PD patients (Figure 1A) using WB analysis. Our results revealed that TWEAK levels were significantly increased in PD patients as compared with non-PD controls. To further confirm the presence of TWEAK in PD serum, an ELISA analysis was performed. Consistent with WB data, ELISA analysis revealed elevated levels of TWEAK in the serum of PD patients as compared with healthy controls (non-PD) (con: 417.4 ± 196.7 pg/mL versus PD: 1243.8 ± 174.3 pg/mL, *p* < 0.001) (Figure 1B), suggesting that TWEAK may be a potential serum protein biomarker for PD.

### 4.2. Oxidative Stress Mechanisms and Mitochondrial Impairment as well as PKCδ and STAT3 Activation Are Augmented in TWEAK-Treated U373 Astrocyte Cells

**Figure 2:**
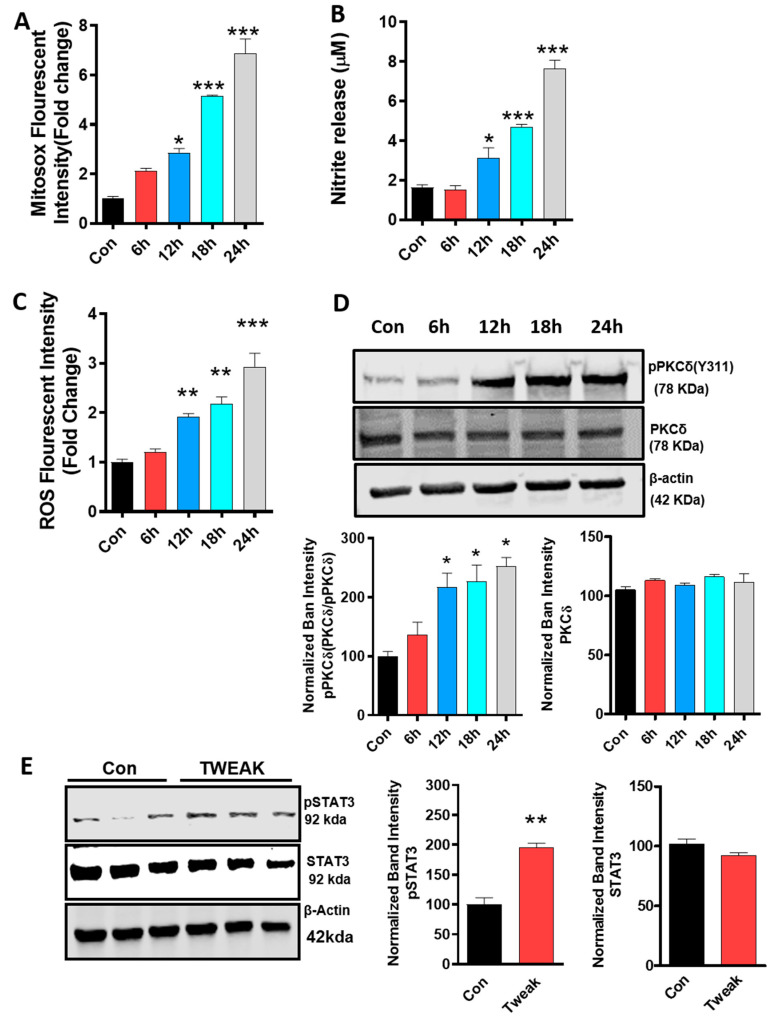

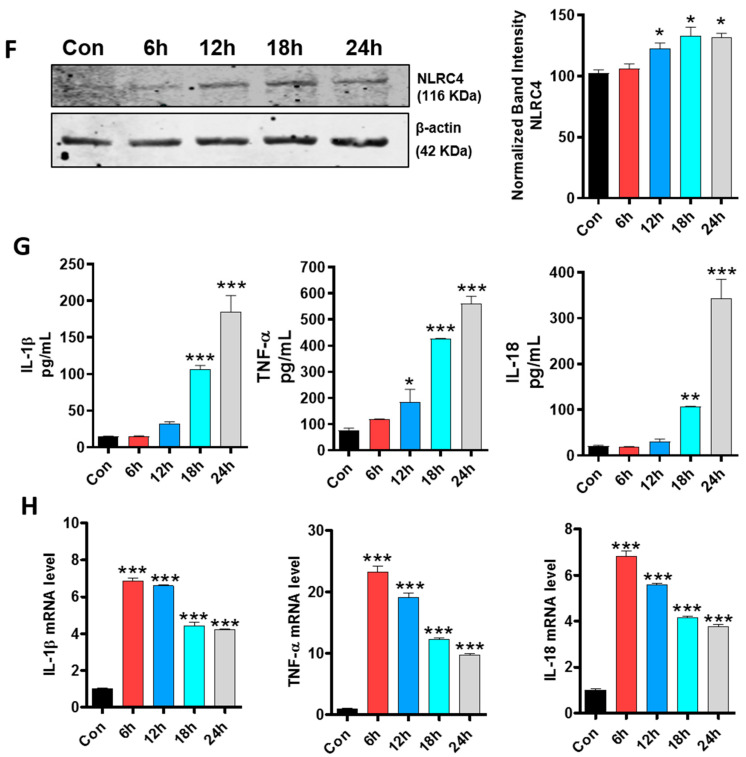
TWEAK-induced oxidative stress response and PKCδ and NLRC4 inflammasome activation concomitant with induction of proinflammatory markers in human astrocyte (U373) cells. (A-H) Human astrocyte (U373) cells were treated with TWEAK (100 ng/mL) for increasing time points (6 h, 12 h, 18 h and 24 h) and analyzed thereafter to evaluate the oxidative stress response. All immunoblots shown in this figure used β-actin as the loading control. (**A**) A MitoSox assay was performed by incubating U373 cells with 5 µM MitoSox dye for 20 min post-TWEAK treatment, and the magnitude of mito ROS was quantified using a fluorescence microplate reader. MitoSox assay shows a time-dependent increase in the level of mitochondrial superoxide post-TWEAK treatment. Data shown are the mean ± SEM from at least three independent experiments. (**B**) Nitrite release assay showing a time-dependent increase in the level of nitric oxide post-TWEAK treatment as determined using the Griess reagent. Data shown are the mean ± SEM from at least three independent experiments. (**C**) ROS generation was measured using the redox-sensitive dye CM-H2DCFDA (1 µM). ROS levels were significantly increased in a time-dependent manner post-TWEAK treatment, as demonstrated by DCF DA fluorescence plate reader analysis. Data shown are the mean ± SEM from at least three independent experiments. (**D**) Cell lysates post-TWEAK treatment were subjected to Western blot analyses for PKCδ and pPKCδ (Y311). Representative blots and densitometric evaluation show a time-dependent increase in PKCδ phosphorylation, indicative of activation post-TWEAK treatment, whereas the expression levels of parent PKCδ were comparable between TWEAK- and vehicle-treated U373 cells. Data shown are the mean ± SEM from at least three independent experiments. (**E**) WB analysis of STAT3 activation in U373 cells treated with TWEAK for 24 h. Representative immunoblots reveal a marked increase in *p*-STAT3 expression levels, but not STAT3 levels, in TWEAK-treated cells. Band quantification using densitometric scanning analysis demonstrated elevated expression of *p*-STAT3 in TWEAK-treated cells as compared to controls. The immunoblot is representative of at least three independent experiments. (**F**) WB analysis for NLRC4 inflammasome in cells stimulated with TWEAK (100 ng/mL) for the indicated time points. Representative immunoblots and band quantification using densitometric scanning analysis revealed a time-dependent increase in NLRC4 inflammasome expression post-TWEAK treatment. Data shown are the mean ± SEM from at least three independent experiments. (**G**) Luminex ELISA analysis of cell culture media post-TWEAK treatment indicating a time-dependent increase in the production of the pro-inflammatory cytokines TNF-α, IL-1β and IL-18 as compared to control cells. Data shown are the mean ± SEM from at least three independent experiments. (**H**) SYBR Green real-time quantitative PCR analysis quantifying mRNA levels of pro-inflammatory markers, namely TNF-α, IL-1β and IL-18 at indicated time points. The normalized increase in gene expression over the control was calculated by the ΔΔCt method. The mRNA level for cytokine expression showed a profound increase at 6 h post-TWEAK treatment. Data shown are the mean ± SEM from at least three independent experiments. Data were analyzed using one-way ANOVA followed by Bonferroni’s post hoc analysis. Asterisks (*** *p* < 0.001, ** *p* < 0.01 and * *p* < 0.05) indicate significant differences between control and treatment groups.

TWEAK has been shown to induce oxidative stress through the aberrant generation of ROS [56] and is actively involved in the progression of the inflammation process [57]. Previous studies from our lab and others have demonstrated a positive correlation between ROS generation, mitochondrial dysfunction and the microglial activation response to diverse inflammagens [39,58]. However, the influence of TWEAK on astroglial oxidative stress and mitochondrial dysfunction is not yet well understood. Therefore, in the present study, we investigated the role of TWEAK in mitochondrial function and oxidative stress with human U373 astrocytes. In the initial set of studies, we determined whether recombinant TWEAK could induce cell death in U373 cells as determined using MTS assay, whereby the percentage of dead cells was assessed in the presence or absence of TWEAK in U373 astrocytes. Consistent with a previous report, 100 ng/mL TWEAK failed to elicit cell death in U373 human astrocytic cells (Figure S1A) [38]. Thus, based on our cell viability studies showing a lack of toxicity, together with other reports [38,59,60] showing that 100 ng/mL TWEAK elicits a proinflammatory response in diverse cell culture models, we utilized this dosing regimen to investigate the TWEAK-induced astroglial activation response for our remaining studies. The U373 astrocytic cells were treated with 100 ng/mL TWEAK for the indicated durations (6, 12, 18, 24 h), and then ROS and mitochondrial (mito)ROS generation were determined by DCFDA and MitoSOX fluorescence plate reader assay, respectively. Concurrently, nitrite release was assayed in the cell culture media using Griess assay. As compared with vehicle-treated cells, TWEAK significantly increased the generation of ROS and mitoROS, as well as nitrite release in a time-dependent manner (Figure 2A–C). Taken together, our studies are consistent with previous studies demonstrating that TWEAK impairs mitochondrial function and enhances the oxidative stress response in diverse cell types, including astrocytes [60,61]. 

Previously, we demonstrated that PKCδ is a key factor in the microglial activation response following exposure to proinflammatory stressors such as α-synuclein, TNF-α and lipopolysaccharide (LPS) [39]. Further highlighting the pivotal role of this enzyme in the heightened neuroinflammatory response evidenced in neurodegenerative disease conditions, PKCδ is abundantly expressed in the ventral midbrain microglial cells of PD patients as compared with age-matched control patients [39]. Furthermore, PKCδ has been implicated in bradykinin receptor-mediated PI turnover in astrocytes, as well as in ammonia-induced astrocyte activation [62,63]. To better understand the mechanisms underlying TWEAK-induced mitoROS and global ROS generation, we examined the activation pattern of the redox-sensitive kinase PKCδ in TWEAK-treated astrocytic cells. Using WB analysis, we determined the time-dependent changes in PKCδ activation in TWEAK-treated U373 cells by examining the extent of PKCδ Tyr 311 phosphorylation, a marker of activation. In cells exposed to TWEAK, a significant increase in PKCδ phosphorylation at site Tyr 311 was evidenced starting at 12 h and remained elevated for the remainder of the treatment period (Figure 2D). Furthermore, densitometric scanning analysis confirmed pronounced activation of PKCδ in TWEAK-treated cells. As expected, the magnitude of parent PKCδ expression was comparable to vehicle-treated cells. Likewise, our immunoblot analyses of PKCδ in TWEAK-stimulated U373 cells at low concentrations (1–50 ng/mL) indicated a significant increase in PKCδ activation at 50 ng/mL further validating the physiological relevance of our findings (Figure S2). Our results are in line with a previous finding from our lab showing PKCδ’s time-dependent activation in LPS-primed, rotenone-stimulated microglial cells [25]. These results imply that PKCδ activation contributes to the astrocyte activation response via impaired mitochondrial function in TWEAK-treated U373 cells. 

The transcription factor signal transducer and activator of transcription 3 (STAT3) is a key signaling mediator that regulates proinflammatory cytokine expression in response to LPS treatment [64]. Additionally, the STAT3 pathway has been shown to contribute to inflammation-associated astrogliosis [65,66,67,68]. ROS production serves as a trigger for STAT3 activation [69], and the redox-sensitive kinase PKCδ is also involved in the phosphorylation of STAT3 in response to IL-6 exposure [70]. We first examined whether TWEAK induced the activation of STAT3 by determining the magnitude of pSTAT3 (Ser727) expression levels in U373 cells treated with TWEAK or vehicle (1% DMSO) for 24 h. WB analysis carried out using anti-pSTAT3 (Ser727) and anti-STAT3 antibodies indicated that TWEAK markedly increased pSTAT3 phosphorylation at site Ser727 in U373 cells (Figure 2E), while total STAT3 protein expression levels remained unchanged between all experimental groups. Thus, our study suggests that STAT3 activation may be regulated by an oxidative stressor, possibly mitoROS. Taken together, our studies indicate that STAT3 activation is associated with TWEAK-induced astroglial dysregulation in U373 cells.

### 4.3. TWEAK Induces NLRC4 Inflammasome Activation in U373 Astrocyte Cells.

It has been demonstrated that NLRC4 can activate pro-caspase-1 via its CARD domain or through an adaptor protein ASC containing a CARD domain, thereby promoting proteolytic cleavage of pro-interleukin-1B (pro-IL-1B) and pro-IL-18 and secretion of mature cytokines that elicit an inflammatory response in response to diverse inflammagens [71]. Moreover, NLRC4 inflammasome activation in the presence of pathogens and cellular damage signals leads to caspase-1-mediated activation of the proinflammatory cytokines IL-1β and IL-18, thereby leading to tissue damage and pyroptosis [72,73]. NLRC4 inflammasome activation is regulated at multiple levels including posttranscriptional modification. In this context, PKCδ has been implicated in NLRC4 inflammasome activation in pathogen-exposed macrophages [74]. As with the PKCδ activation shown above in TWEAK-stimulated astrocytes, we reasoned that the NLRC4 inflammasome may be activated in response to TWEAK in U373 cells. For this purpose, we determined the temporal pattern of NLRC4 activation at 6, 12, 18 and 24 h post-TWEAK (100 ng/mL) treatment using WB analysis. Time course studies revealed a persistent increase in NLRC4 activation starting at 12 h and remaining elevated for the remainder of the treatment duration, as compared with controls (Figure 2F). NLRC4 inflammasome assembly leads to the generation of proinflammatory cytokines, namely IL-1β and IL-18, via a caspase-1-dependent mechanism [75], so we determined the magnitude of proinflammatory cytokine release, including TNF-α, IL-1β and IL-18. Our Luminex ELISA analysis revealed that TWEAK induced a significant time-dependent increase in proinflammatory cytokine release in U373 cells as compared to vehicle-treated cells (Figure 2G). The extent of astrocyte activation upon TWEAK treatment was further determined by examining the mRNA levels of the aforementioned proinflammatory cytokines, which we found to be elevated in TWEAK-treated U373 cells as compared to vehicle-treated cells (Figure 2H). Additionally, upon stimulation of U373 cells with low concentrations of TWEAK (1–50 ng/mL) (Figure S2), a significant increase in NLRC4 inflammasome expression and its activation markers were evidenced at 50 ng/mL, further validating the ability of TWEAK to induce proinflammatory signaling. Thus, it is conceivable that NLRC4 inflammasome activation contributes to the generation of proinflammatory cytokines, including TNF-α, IL-1β and IL-18, during TWEAK-induced astrocyte activation via a PKCδ-dependent mechanism in U373 cells [74].

### 4.4. TWEAK Stimulation of Primary Astrocytes Is Associated with the Activation of PKCδ, NLRC4 and STAT3, as well as the Generation of Proinflammatory Cytokines

**Figure 3:**
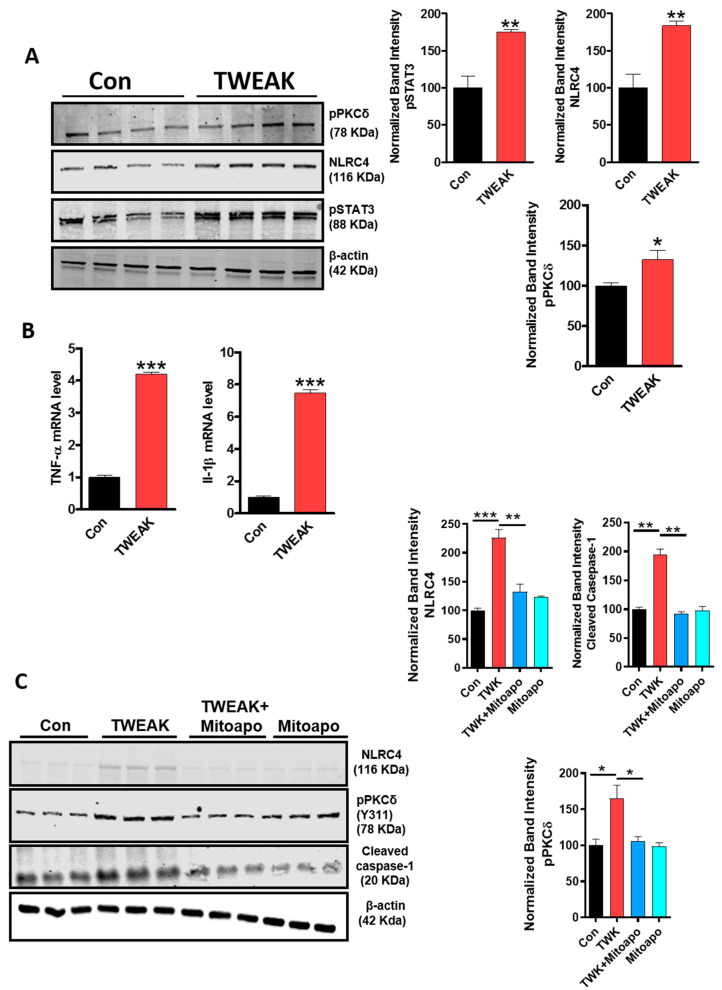
The NLRC4 inflammasome and PKCδ activation positively correlate with STAT3 activation and the generation of proinflammatory cytokines in TWEAK-stimulated primary astrocytes. (**A**) Primary astrocytes were stimulated with TWEAK (100 ng/mL) for 24 h, and cell lysates were assessed for PKCδ, NLRC4 and STAT3 by immunoblotting analysis; β-actin was used as the internal loading control. Quantification of bands using densitometric scanning analysis revealed that TWEAK promoted the activation of the aforementioned inflammasome-related proinflammatory markers. Data presented as mean ± SEM and representative of four independent experiments. (**B**) Primary astrocytes were subjected to real-time quantitative PCR analysis to quantify mRNA levels of pro-inflammatory markers, namely TNF-α, IL-1β and IL-18, at indicated time points. The normalized fold increase in gene expression over the control was calculated by the ΔΔCt method. Primary astrocyte cells were pretreated with mito-apocynin (10 µM) for 1 h and analyzed for target protein expression levels 24 h post-TWEAK treatment. (**C**) Cell lysates were analyzed for pPKCδ, NLRC4 and cleaved-caspase-1 using Western blot analysis. Representative immunoblots and quantification of bands using densitometric scanning analysis are depicted. Mito-apocynin (10 µM) pretreatment yielded a significant reduction in protein levels of NLRC4 inflammasome markers, as assessed by WB. Data are presented as the mean ± SEM and representative of four independent experiments. Data were analyzed using one-way ANOVA followed by Bonferroni’s post hoc analysis. Asterisks (*** *p* < 0.001, ** *p* < 0.01 and * *p* < 0.05) indicate significant differences between control and treatment groups.

The findings in our U373 astrocytic cell line led us to compare TWEAK-induced inflammasome activation and associated proinflammatory-signaling markers in primary astrocytes. Astrocytes isolated from postnatal pups (0–2 d postnatal) were utilized for the biochemical experiments detailed below. First, we determined the effects of TWEAK on the activation of PKCδ, STAT3 and the NLRC4 inflammasome in primary astrocytes in culture. As expected, the protein expression levels of the aforementioned proinflammatory markers were elevated in TWEAK-stimulated primary astrocytes as compared to vehicle-treated controls as assessed via WB (Figure 3A). The effects of TWEAK on mRNA levels of proinflammatory cytokines, including TNF-α and IL-1β, were then examined by qPCR, which found that their mRNA levels increased in TWEAK-stimulated primary astrocytes as compared to controls (Figure 3B). We then examined the effects of mito-apocynin on the expression of proinflammatory markers, including PKCδ, NLRC4 and caspase-1 in TWEAK-stimulated primary astrocytes. Western blot analysis showed that mito-apocynin treatment attenuated TWEAK-induced PKCδ and NLRC4 inflammasome activation markers in primary astrocytes (Figure 3C). These findings clearly demonstrate the positive association between the mitochondrial oxidative stress response and activation of PKCδ, STAT3 and the NLRC4 inflammasome in promoting TWEAK-induced astrocyte activation. These findings support the possibility that the increased gene expression of TNF-α and IL-1β evidenced following TWEAK treatment of primary astrocytes may be partly mediated via a PKCδ-dependent mechanism, thereby leading to pronounced astroglial reactivity.

### 4.5. TWEAK-Induced ROS Generation, NLRC4 Inflammasome Activation and STAT3 Activation Are Regulated by PKCδ in U373 Cells

**Figure 4:**
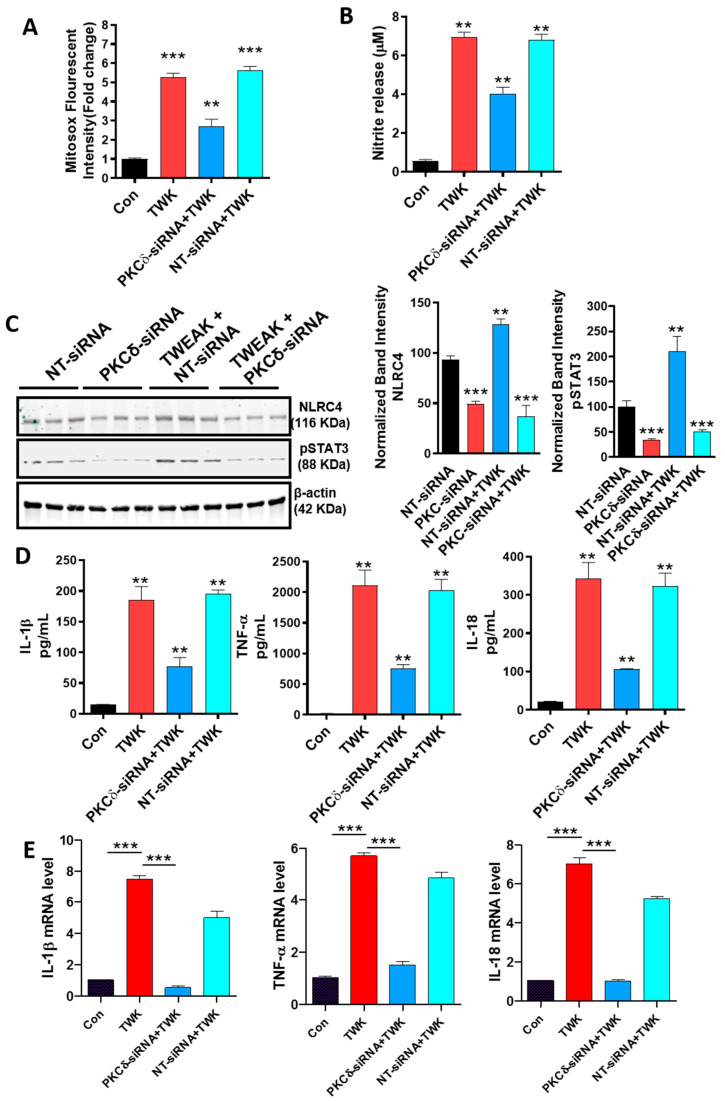
PKCδ regulates TWEAK-induced ROS generation, nitrosative stress and STAT3 and NLRC4 inflammasome activation in TWEAK-treated human astrocytic U373 cells. (**A**–**E**) Following transfection with control siRNA or PKCδ siRNA for 48 h, human astrocyte (U373) cells were treated with TWEAK (100 ng/mL) for 24 h. (**A**) MitoSox assay was conducted by incubating U373 cells with 5 µM MitoSox dye for 20 min; the results were quantified using a fluorescence microplate reader. PKCδ specific knockdown significantly reduced the generation of mitochondrial superoxide in TWEAK-treated U373 cells. Data shown are the mean ± SEM from at least three independent experiments. (**B**) Nitric oxide production in cell supernatant was determined using Griess assay post-TWEAK treatment. Nitric levels were significantly reduced in PKCδ siRNA transfected cells as compared to scrambled siRNA transfected cells exposed to TWEAK. (**C**) Cell lysates were analyzed for the NLRC4 and pSTAT3 using Western blot analysis. Representative immunoblots and band quantification using densitometric scanning analysis revealed that siRNA-mediated gene silencing of PKCδ diminished NLRC4 inflammasome activation and STAT3 phosphorylation post-TWEAK treatment. Data shown are the mean ± SEM from at least three independent experiments. Collectively, siRNA mediated PKCδ silencing led to a significant reduction in the oxidative stress mechanism and mitochondrial dysfunction in TWEAK-stimulated U373 cells. Data shown are the mean ± SEM from at least three independent experiments. (**D**) Cytokines levels including TNF-α, IL-1β and IL-18 secretion were quantified in cell supernatants using ELISA. Data shown are the mean ± SEM from at least three independent experiments. (**E**) PKCδ siRNA transfected cells were harvested post-TWEAK treatment, and SYBR Green real-time quantitative PCR was done to quantify the mRNA level of pro-inflammatory markers, including TNF-α, IL-1β and IL-18. Data shown are the mean ± SEM from at least three independent experiments. The normalized increase in gene expression over the control was calculated by the ΔΔCt method. The mRNA levels of NLRC4 inflammasome related markers including IL-1β and IL-18, as well as TNF-α, were significantly decreased in PKCδ siRNA transfected cells that were treated with TWEAK, as determined by ELISA and qPCR studies. Data were analyzed using one-way ANOVA followed by Bonferroni’s post hoc analysis. Asterisks (*** *p* < 0.001, ** *p* < 0.01) indicate significant differences between control and treatment groups.

Previously, we have shown that the redox-sensitive kinase PKCδ is a critical determinant of the microglial activation response [25,39]. However, the link between PKCδ activation, ROS generation and mitochondrial dysfunction during the astroglial proinflammatory signaling response remains poorly characterized. To gain further insight into the mechanism by which TWEAK modulates mitochondrial dysfunction and the oxidative stress response, we determined the contribution of PKCδ in ROS generation and mitochondrial dysfunction utilizing an siRNA targeted against PKCδ. To accomplish this, U373 cells were transfected with either a scrambled siRNA or an siRNA against PKCδ for 48 h to downregulate PKCδ expression levels. In the initial set of experiments, we verified the efficiency of PKCδ knockdown (KD) by using WB and densitometric analyses to show (Figure S3) that siRNA-mediated gene silencing of PKCδ markedly reduced endogenous PKCδ levels (60–70%) in U373 cells. We next examined the impact of RNAi-mediated gene depletion of PKCδ on the TWEAK-induced oxidative stress response in U373 cells. In line with previous findings from our group and others that PKCδ inhibition could reduce mitochondria-dependent oxidative and nitrosative stress responses in diverse experimental models of inflammation [39,42,76], the generation of mitoROS and nitrite species was significantly decreased in PKCδ siRNA-transfected cells as compared to scrambled siRNA-transfected cells exposed to TWEAK (Figure 4A,B). Thus, these data indicate that PKCδ is a critical regulator of mitochondrial oxidative and nitrosative stress responses, thereby implicating its possible involvement in the deleterious, PD-associated astrocytic neuroinflammatory response and the associated DAergic neurodegenerative response. 

To further investigate the impact of PKCδ on inflammasome activation, we examined the magnitude of expression of the NLRC4 inflammasome and its related activation markers using WB and ELISA analysis. Our use of siRNA-mediated gene silencing of PKCδ dramatically reduced NLRC4 inflammasome activation (Figure 4C) with a concomitant reduction in mRNA expression and secretion of proinflammatory cytokines, including TNF-α, IL-1β and IL-18, as compared to scrambled siRNA-transfected cells following TWEAK treatment (Figure 4D,E), demonstrating that PKCδ is a critical upstream regulator of the NLRC4 inflammasome activation mechanism. Indeed, our findings are consistent with a previous finding that PKCδ is a critical upstream regulator of NLRC4 inflammasome activation [74]. To further substantiate PKCδ’s contribution to the TWEAK-induced oxidative stress response, we examined the influence of RNAi-mediated KD of PKCδ on STAT3 tyrosine phosphorylation in TWEAK-treated cells. For this purpose, we assayed the protein expression profile of STAT3 and STAT3 phosphorylation in TWEAK-treated U373 cells transfected with or without PKCδ siRNA. Our WB analysis showed that TWEAK treatment increased the phosphorylation of STAT-3 (Y-727) in U373 astrocytes (Figure 4C), and that suppressing PKCδ activity via siRNA-mediated gene silencing inhibited TWEAK-induced STAT3 phosphorylation. Collectively, these results demonstrate the involvement of PKCδ during the oxidative stress response and STAT3 serine phosphorylation, as well as NLRC4 inflammasome activation during TWEAK-induced astroglial reactivity.

### 4.6. Pharmacological Inhibition of Mitochondrially-Derived ROS via Mito-Apocynin Suppresses Mitochondrial Impairment with a Concomitant Reduction in PKCδ and NLRC4 Inflammasome Activation as well as STAT-3 Activation in Astrocytes

**Figure 5:**
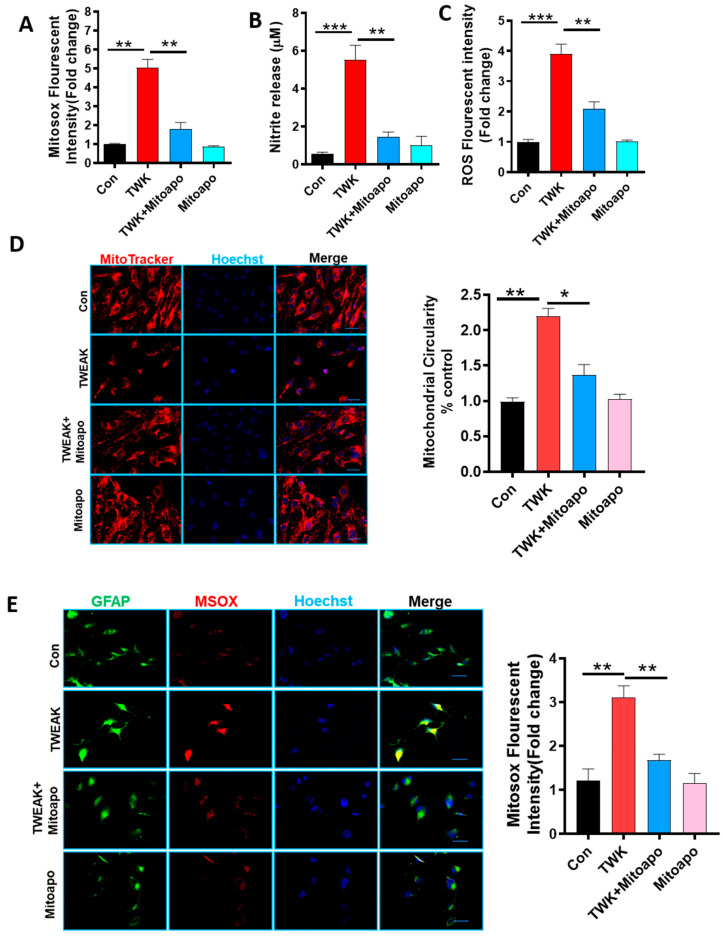

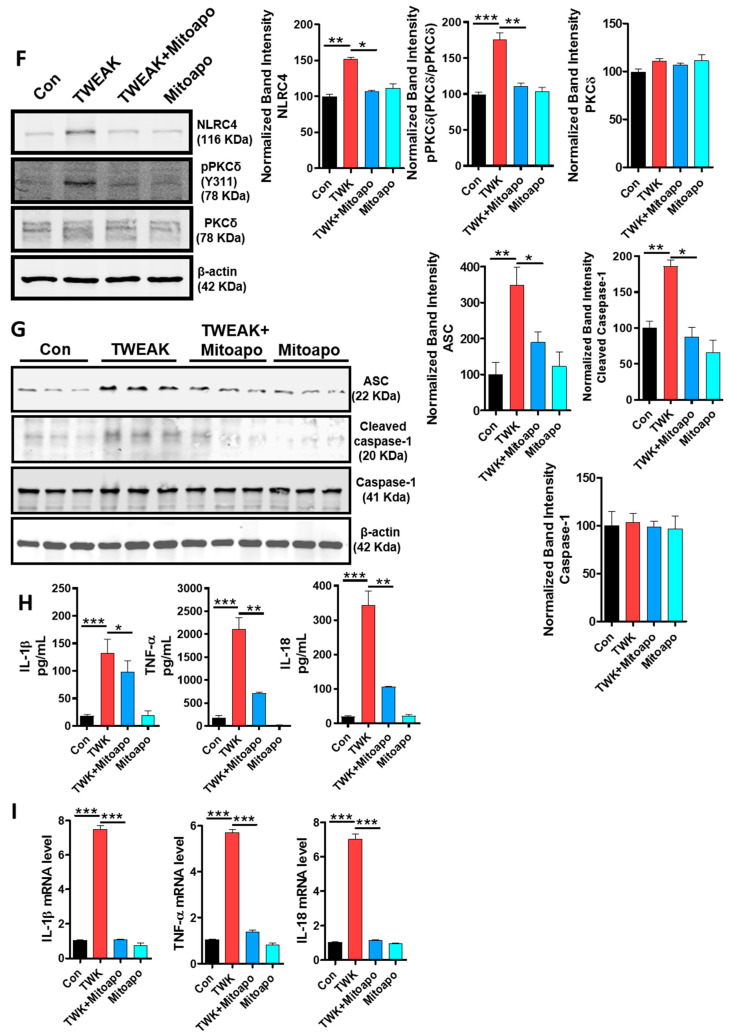

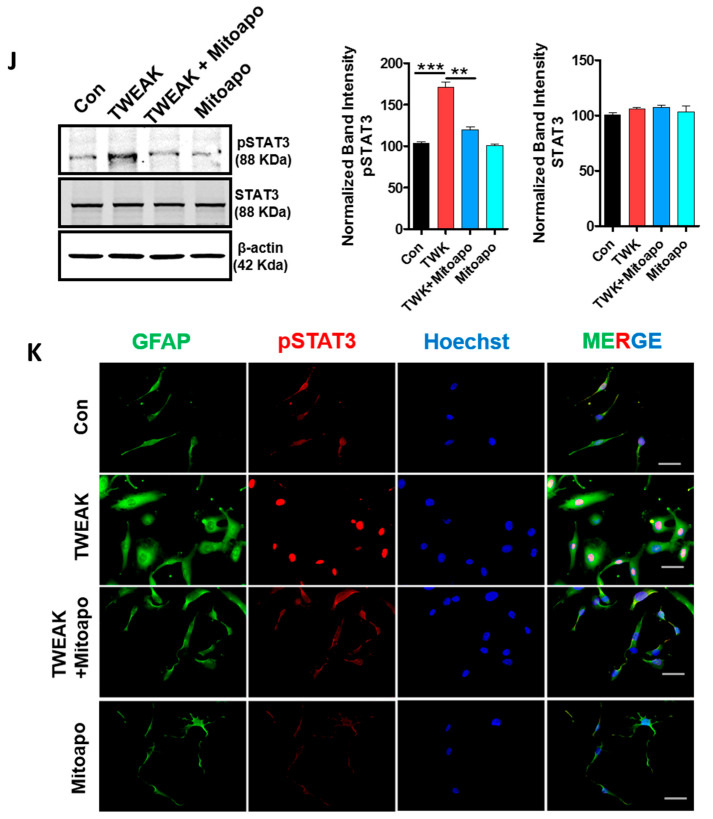
Inhibition of mitochondrial oxidative stress via Mito-apocynin attenuates TWEAK-induced, mitochondria-derived oxidative stress response and nitrosative stress, as well as PKCδ/NLRC4 and STAT3 activation in U373 cells. (**A**–**E**) Human astrocyte (U373) cells were pretreated with mito-apocynin (10 µM) for 1 h, and diverse oxidative stress analyses were performed 24 h post-TWEAK treatment. (**A**) MitoSox assay was conducted by incubating the U373 cells with MitoSox dye for 20 min, and the extent of mito ROS was assessed via fluorescence microplate reader. Mito-apocynin significantly reduced TWEAK-induced mitochondrial superoxide levels. Data shown are the mean ± SEM from at least three independent experiments. (**B**) Nitric oxide levels in the supernatant were determined using the Griess assay. Mito-apocynin significantly reduced TWEAK-induced nitrite species generation. Data are expressed as the mean ± SEM from at least three independent experiments. (**C**) ROS generation was measured using the redox-sensitive dye CM-H2DCFDA (1 µM), and the magnitude of ROS generation was assessed via fluorescence plate reader analysis. Mito-apocynin reduced TWEAK-induced ROS generation. (**D**) MitoTracker assays were implemented to assess changes in mitochondrial morphology in U373 cells. The circularity analysis was indicative of mitochondrial fragmentation, which was significantly elevated in TWEAK-treated cells and ameliorated by mito-apocynin pretreatment. Data are shown as the mean ± SEM from at least three independent experiments. Scale bar = 20 µm. (**E**) Representative immunofluorescent images show that mito-apocynin pretreatment attenuates TWEAK-induced generation of mitochondrial ROS. Orange/yellow fluorescence, which is indicative of increased generation of mitoROS, was pronounced in TWEAK-stimulated U373 cells. Scale bar = 20 µm. (**F**–**I**) Human astrocyte (U373) cells were pretreated with mito-apocynin (10 µM) for 1 h and analyzed thereafter post-TWEAK treatment for 24 h. (**F**,**G**) Cell lysates were analyzed for PKCδ, pPKCδ and NLRC4 inflammasome markers, as well as NLRC4, ASC, cleaved-caspase-1 and caspase-1 using Western blot analysis. Representative immunoblots and the quantification of bands using densitometric scanning analysis are depicted. Mito-apocynin (10 µM) pretreatment shows a significant reduction in protein levels of NLRC4 inflammasome markers, as assessed by WB. Data are shown as the mean ± SEM from at least three independent experiments. (**H**) Cytokine levels including TNF-α, IL-1β and IL-18 secretion (NLRC4-related markers) were quantified in cell supernatants using ELISA. Data are shown as the mean ± SEM from at least three independent experiments. (**I**) Mito-apocynin (10 µM) pretreated cells were harvested post-TWEAK treatment, and SYBR Green real-time quantitative PCR was done to quantify mRNA levels of pro-inflammatory markers, i.e., TNF-α, IL-1β and IL-18. The normalized increase in gene expression over the control was calculated by the ΔΔCt method. Data are shown as the mean ± SEM from at least three independent experiments. TNF-α, IL-1β and IL-18 level were significantly decreased in mito-apocynin (10 µM) pretreated TWEAK-stimulated cells, as confirmed by our ELISA and qPCR, respectively. U373 astrocytic cells were pretreated with mito-apocynin (10 µM) for 1 h, followed by TWEAK (100 ng/mL) for another 24 h. STAT3 and pSTAT3 expression levels were analyzed by Western blotting analysis. (**J**) Representative immunoblots of STAT3 and pSTAT3. Densitometric quantification of pSTAT3 protein expression levels which were normalized to β-actin showed that mito-apocynin attenuated TWEAK-induced pSTAT3 expression in U373 astrocytes. Data shown are the mean ± SEM from at least three independent experiments. (**K**) U373 Astrocytes were treated with TWEAK (100 ng/mL) for 1 h in the presence or absence of mito-apocynin. For ICC studies, cells were dual-labeled for STAT3 (Red) and GFAP (green) and nuclei were counterstained with Hoechst stain (Blue). Representative immunofluorescent images of U373 cells that were costained for GFAP and pSTAT3 revealed that mito-apocynin inhibited TWEAK-induced nuclear trafficking of pSTAT3. Results represent three independent experiments. Scale bar = 20 µm. Data are shown as the mean ± SEM from at least three independent experiments. Data analysis was performed using two-way ANOVA followed by Bonferroni’s post hoc analysis. Asterisks (*** *p* < 0.001, ** *p* < 0.01 and * *p* < 0.05) indicate significant differences between control and treatment groups.

Mito-apocynin, a mitochondrially targeted antioxidant, has been shown to protect against oxidative damage, glia-mediated inflammation and nigrostriatal neurodegeneration in experimental models of PD [77]. Furthermore, we previously demonstrated that mito-apocynin elicits neuroprotection in a progressive neurodegenerative mouse model by inhibiting glial activation [78]. Having demonstrated that TWEAK promotes mitochondrial impairment via heightened generation of mitochondria-derived ROS, we next investigated whether inhibition of mitoROS generation via mito-apocynin modulated TWEAK-induced PKCδ activation as well as NLRC4 inflammasome and STAT3 activation in U373 astrocytic cells. We found that mito-apocynin pretreatment significantly decreased ROS, nitric oxide and mitochondria superoxide generation in TWEAK-treated U373 astrocytes as compared with TWEAK-treated U373 cells (Figure 5A–C). To further clarify mitochondrial ROS generation, immunofluorescence microscopy was performed to determine ROS distribution within mitochondria and its impact on mitochondrial morphology in TWEAK-treated U373 cells. Our IHC studies further confirmed that mito-apocynin ameliorated the TWEAK-induced mitochondrial impairment (Figure 5D,E), as defined by a marked reduction in the number of damaged (circular) mitochondria. Consistent with this finding, an increase in mitoROS levels, as determined by MitoSOX Red fluorescence, was evidenced in TWEAK-treated U373 cells as compared with controls. Moreover, mito-apocynin treatment markedly inhibited TWEAK-induced mitoROS generation in U373 cells, suggesting that mitoROS contributes to TWEAK-induced astroglial reactivity. 

We then examined the effects of mito-apocynin on the expression of proinflammatory markers, including PKCδ and NLRC4. WB analysis showed that mito-apocynin treatment attenuated TWEAK-induced PKCδ and NLRC4 inflammasome activation (Figure 5F). To further assess the effect of mitoROS generation on TWEAK-induced inflammasome activation markers, we examined the impact of mito-apocynin on TWEAK-induced inflammasome activation markers, including ASC and cleaved caspase-1 protein expression in U373 cells. A WB analysis (Figure 5G) revealed that mito-apocynin markedly attenuated TWEAK-induced inflammasome activation markers in U373 cells, suggesting that mitoROS serves as a critical contributor to astroglial inflammasome activation after TWEAK stimulation. Similar to what we observed (Figure 5G), together with previous reports [77,78], mito-apocynin suppressed the TWEAK-induced upregulation of proinflammatory cytokine (TNF-α, IL-1β, and IL-6) generation, as well as their secretion, as determined by qPCR and ELISA Luminex analysis, respectively (Figure 5H,I). Given that STAT3 is a critical regulator of reactive astrogliosis after spinal cord injury (SCI) and is upregulated during oxidative stress-induced neuronal injury [68,79,80], we next examined whether mito-apocynin suppressed STAT3 activation. We found that treatment with mito-apocynin significantly reduced the levels of TWEAK-induced STAT3 phosphorylation in U373 cells (Figure 5J), as determined by WB. Likewise, mito-apocynin attenuated the TWEAK-induced pSTAT3 nuclear translocation as determined by immunofluorescence analysis (Figure 5K). Together, our findings indicate that mitoROS may act as a positive modulator of astrocyte activation by inducing the PKCδ/STAT3/NLRC4 inflammasome signaling axis, suggesting that mitoROS exerts multiple effects with regards to the regulation of the astrocytic activation response.

### 4.7. Inhibition of TWEAK-Fn14 Signaling Attenuated TWEAK-Induced Proinflammatory Cytokine Generation

**Figure 6:**
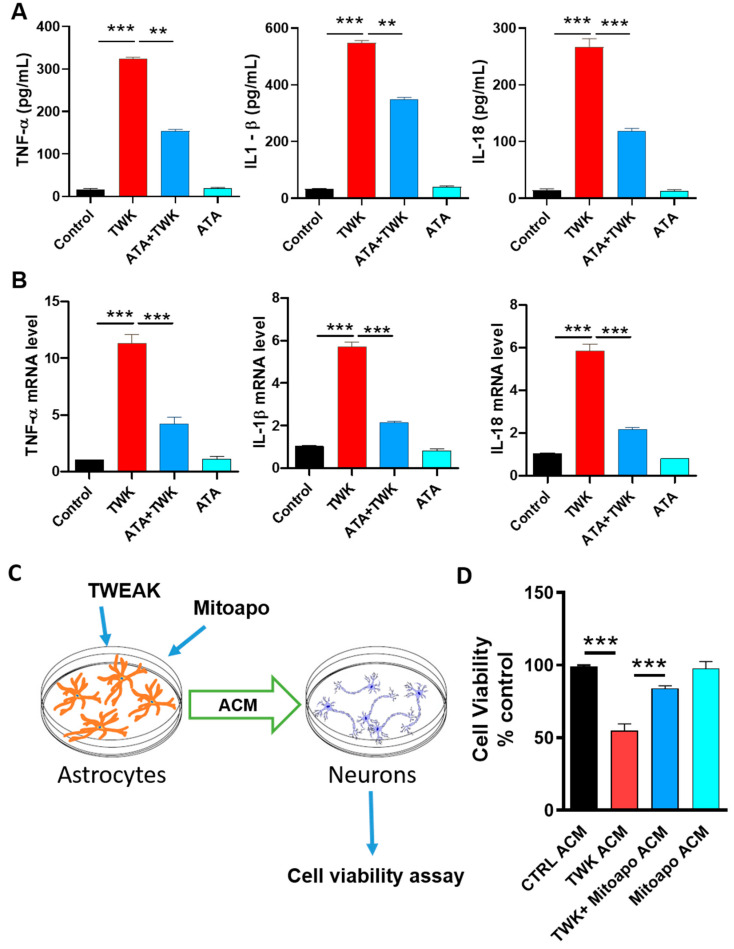
Aurintricarboxylic acid (ATA) attenuates the TWEAK-induced release of pro-inflammatory cytokines and mito-apocynin suppresses TWEAK astrocyte-conditioned media (ACM)-induced DA ergic neurotoxicity. Human astrocyte (U373) cells were pretreated with ATA (10 µM) for 2 h, followed by TWEAK (100 ng/mL) for another 24 h. The cultures were assayed for proinflammatory cytokines using ELISA and qPCR analysis. (**A**) TNF-α, IL-1β and IL-18 secretion (NLRC4-related markers) were quantified in cell supernatants using ELISA. ATA pretreatment significantly reduced the secretion of proinflammatory markers, as indicated by ELISA results. Data shown are the mean ± SEM from at least four independent experiments. (**B**) U 373 cells were subjected to the same treatment conditions as stated above, and mRNA levels of the aforementioned cytokines were analyzed by qPCR. The normalized increase in gene expression over the control was calculated by the ΔΔCt method. Data shown are the mean ± SEM from at least four independent experiments. (**C**,**D**) Mito-apocynin reduced TWEAK ACM-induced dopamin(DA)ergic cell death, as assessed via MTS cell viability assay. U373 cells were treated with TWEAK for 24 h, and subsequently, ACM from TWEAK-treated U373s with or without mito-apocynin was applied to LUHMES human DAergic neuronal cells. A cell viability assay was performed at 12 h after ACM application to LUHMES cells. Data were analyzed using one-way ANOVA followed by Bonferroni’s post hoc analysis. Data shown are the mean ± SEM from at least four independent experiments. Asterisks (*** *p* < 0.001, ** *p* < 0.01) indicate significant differences between control and treatment groups.

In our experiments, TWEAK elicited astroglial activation in U373 cells. To examine whether TWEAK elicits its proinflammatory astroglial activation response via the Fn14 receptor, we examined the effects of Aurintricarboxylic acid (ATA, a specific inhibitor of TWEAK-Fn14 signaling, in U373 cells. Fn14 has been identified as a key receptor mediating the TWEAK-induced proinflammatory signaling response, and in a recent study, ATA blocked TWEAK-Fn14-NFkB-dependent signaling but not TNF-α-TNFR-NF-κB-driven signaling [81], further supporting its specificity. Therefore, we analyzed the effects of ATA on TWEAK-induced proinflammatory cytokine generation by treating U373 cells with 10 µM ATA for 1 h, followed by TWEAK treatment for another 24 h. Following TWEAK treatment, the mRNA and protein expression of proinflammatory cytokines, including TNF-α, IL-1β and IL-18, markedly increased, as assessed via qPCR and ELISA analysis (Figure 6A,B). Indeed, treatment with the specific TWEAK-Fn14 inhibitor diminished TWEAK-induced proinflammatory cytokine generation and release in U373 cells, suggesting that TWEAK-induced proinflammatory signaling occurs via an Fn14-dependent mechanism. Together, our data corroborate the proinflammatory role of TWEAK in U373 astrocytic cells, wherein the TWEAK-Fn14 signaling mechanism is likely to contribute to astrocytic proinflammatory signaling.

### 4.8. Inhibiting Astrocytic Mitochondrial Oxidative Stress Suppressed TWEAK Astrocyte-Conditioned Media (ACM)-Induced Dopaminergic Neurotoxicity

The astroglial activation response and subsequent release of proinflammatory cytokines in the brain could lead to neuronal cell death [82]. After showing that inhibition of mitochondrial oxidative stress via mito-apocynin negates TWEAK-induced proinflammatory signaling by reducing the generation and secretion of proinflammatory cytokines, we reasoned that suppressing astroglial mitochondrial oxidative stress may impact neuronal viability via neuron-astrocyte crosstalk. As depicted in our experimental schematic (Figure 6C), U373 cells were treated with 100 ng/mL TWEAK for 24 h with or without mito-apocynin (10 µM) treatment. ACM collected from astrocytes in culture was subsequently applied for 12 h to LUHMES human DAergic neuronal cells in culture, which has been considered to be physiologically relevant in the study of the mechanisms underlying DAergic neurodegeneration in the context of PD related neuropathology [58,83]. As assessed by MTS assay 12 h following ACM treatment, TWEAK-treated ACM elicited a marked reduction in LUHMES cell viability as compared with vehicle-treated cells. In contrast, LUHMES cells treated with ACM from mito-apocynin pretreated TWEAK-stimulated U373 cells (Figure 6D) exhibited a dramatic reduction in TWEAK-induced DAergic neurotoxicity. These results indicate that TWEAK-stimulated astrocytic cells likely secrete neurotoxic mediators via a mitochondrial oxidative stress mechanism, thereby contributing to DAergic neuronal loss. In line with our findings, Wu et al. [84] reported that reducing ROS generation via salidroside improved viability in a neuron-astrocyte co-culture model. 

### 4.9. MPTP-Induced Astrocyte Activation Involves the Upregulation of TWEAK with Concomitant NLRC4 Inflammasome Activation and TH Neuronal Loss

**Figure 7:**
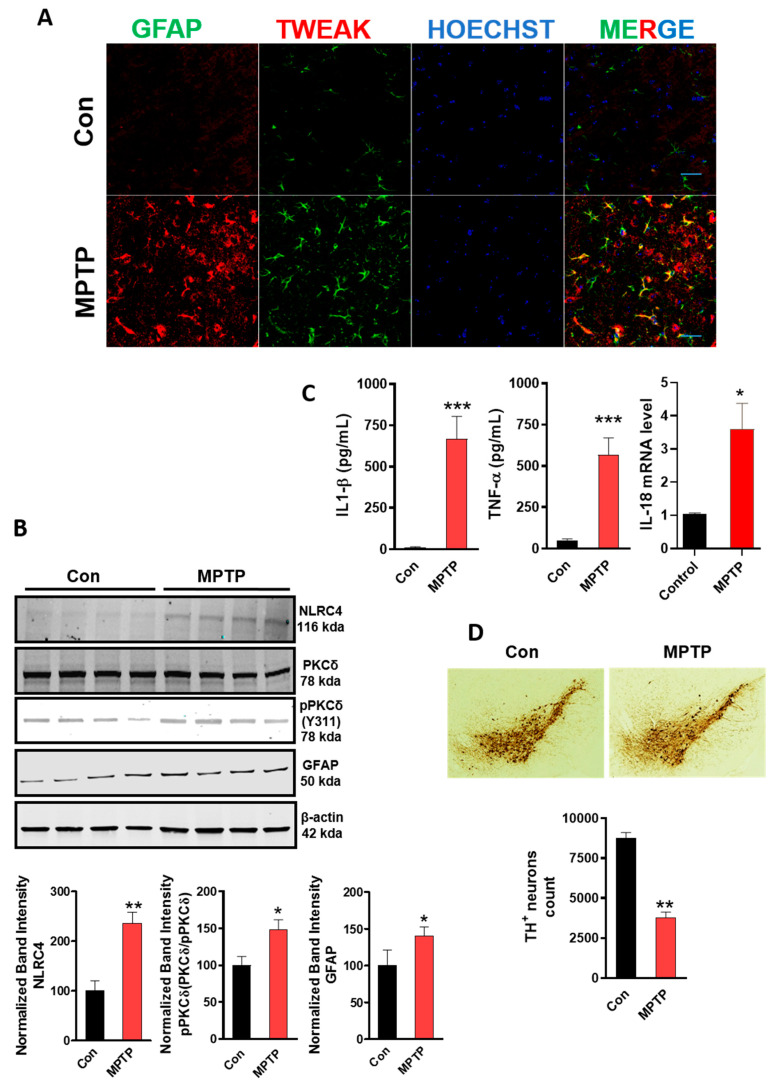

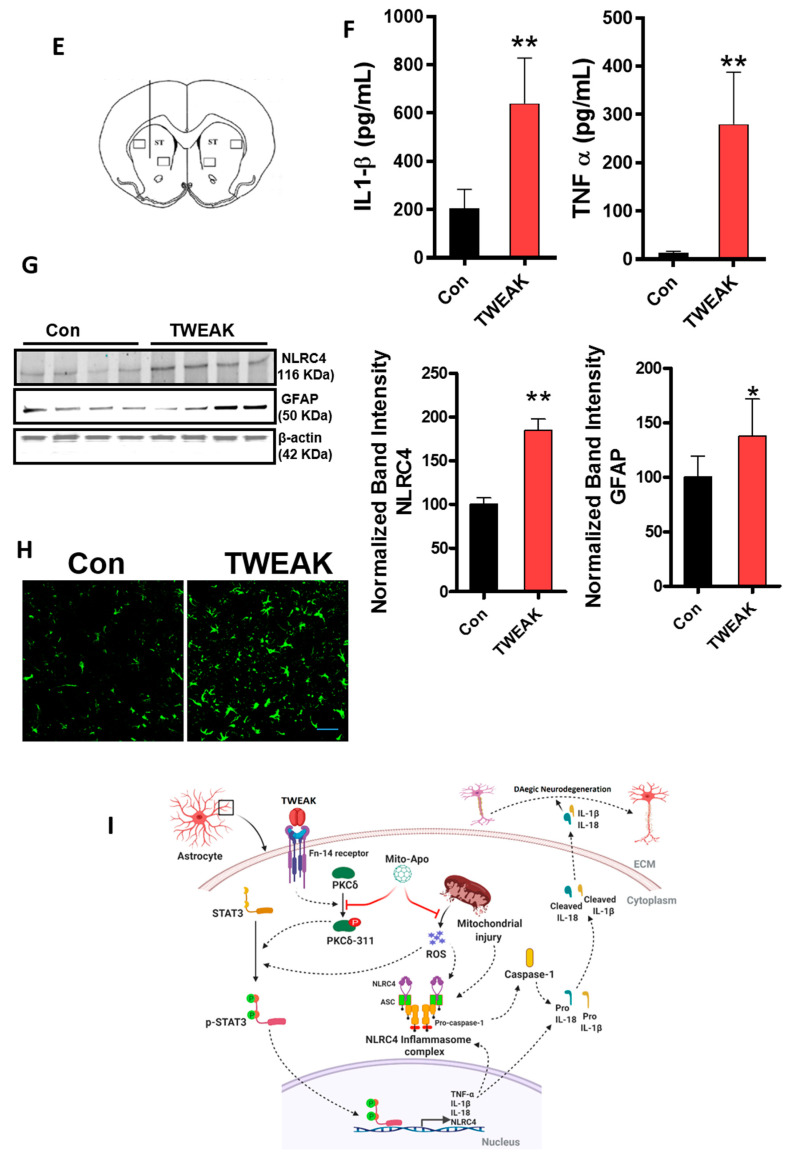
Enhanced TWEAK expression within activated astrocytes positively correlates with NLRC4 inflammasome activation and TH neuronal loss in the nigra of MPTP-treated mice. A similar response was evidenced in the ventral midbrain (SNpc) of mice that received an intrastriatal TWEAK injection. (**A**–**D**) Mice were intraperitoneally injected with MPTP (25 mg/kg) or PBS. Upon sacrifice, mouse brain tissues were harvested and used for IHC, WB and ELISA analysis. (**A**) Colocalization of TWEAK and GFAP, as assessed via IHC analysis, showed increased astrocyte activation and colocalization of TWEAK in the MPTP mice as compared to the PBS-injected mice. Scale bars = 20 µm. (**B**) Representative immunoblots illustrating the expression of NLRC4, PKCδ, pPKCδ and GFAP in the substantia nigra (SN) of MPTP- and saline-treated mice. Immunoblotting analysis of SN tissue lysates from mice treated with MPTP or PBS. Bottom, quantification of the aforementioned proteins using densitometric scanning analysis; β-actin was used as a loading control. Data shown are the mean ± SEM from at least four independent experiments. (**C**) Increased generation of the proinflammatory cytokines TNF-α, IL-1β and IL-18 was observed in the MPTP-administered mice as compared to PBS-injected mice, as determined by Luminex cytokine and qPCR analysis. Data shown are the mean ± SEM from at least four independent experiments. (**D**) Mice were subjected to the same treatment conditions as mentioned above, and tyrosine hydroxylase (TH) levels were assessed using 3,3′-diaminobenzidine (DAB) immunostaining in the SN of MPTP- and saline-treated mice. Representative photomicrographs from coronal midbrain sections containing TH-positive neurons showed a dramatic reduction of TH staining in MPTP-treated mice as compared to saline-treated mice; this was further confirmed by unbiased stereological counts of TH positive neurons in the SNpc brain region. Data are the mean ± S.E.M; *n* = 4 independent mice. (**E**) Representational diagram depicting intrastriatal TWEAK injection in mice. (**F**) Mice were stereotaxically injected with 2 µL of either PBS or TWEAK (1 µg/µL) into the left striatal brain region. TWEAK-infused mice displayed increased levels of proinflammatory cytokines, including TNF-α and IL-1β, as compared to PBS-infused mice, as determined by Luminex ELISA analysis. (**G**) Tissue lysates from the striatum of mice injected with TWEAK or PBS were analyzed for NLRC4 and GFAP in Western blots. Representative immunoblots of NLRC4 and GFAP and densitometric quantification revealed increased protein expression of NLRC4 and GFAP in the nigra of TWEAK-infused mice as compared to PBS-infused mice. (**H**) Representative immunohistochemical (IHC) images of GFAP show increased expression of GFAP in TWEAK-infused mice as compared to the PBS-injected mice. Scale bars = 20 µm. Data are the mean ± SEM from at least four independent mice. Data were analyzed using a two-tailed t-test. Asterisks (*** *p* < 0.001, ** *p* < 0.01 and * *p* < 0.05) indicate significant differences between control and treatment groups. (**I**) Schematic illustration of the proposed mechanism of TWEAK-induced astroglial activation response and potential impact on DAergic neuronal integrity. Each solid arrow represents a step in the astrocytic proinflammatory activation pathway. TWEAK binds to the TWEAK-Fn14 receptor and activates STAT3 and NLRC4 through PKCδ and a mitochondrial oxidative stress-dependent mechanism. PKCδ activation and mitochondrial oxidative stress lead to STAT3 phosphorylation and sequential nuclear translocation, and then promote proinflammatory cytokine generation. Finally, IL-1β and IL-18 are released into the extracellular environment, possibly via an NLRC4 inflammasome activation mechanism. This release of a proinflammatory neurotoxic mediator leads to a loss of DAergic neurons in the PD environment. Mitoapocynin inhibits the TWEAK-induced PKCδ signaling axis, as well as the mitochondrial oxidative stress response.

After establishing that TWEAK induces dysregulation of astroglial function using both a well-established U373 human astrocytic cell line and primary mouse astrocytes in culture, we investigated whether TWEAK was upregulated in an MPTP mouse model of PD. We and others [77,85] have demonstrated that the MPTP mouse model is a reliable in vivo model that displays a robust astrocytic neuroinflammatory response. Thus, in the present study, we investigated the association between astrocytic TWEAK upregulation, NLRC4 inflammasome activation and TH neuronal loss in the SN of MPTP-treated mice. Mice were exposed to MPTP for five consecutive days (25 mg/kg, i.p.) and sacrificed after seven days, following the last dose of MPTP. Consistent with a previous report showing that TWEAK is inducible under disease conditions [86], it was localized to activated astrocytes, concomitant with the increased GFAP protein expression indicative of astrogliosis in the SN of MPTP-treated mice (Figure 7A). Moreover, this increase coincided with increased expression of PKCδ and NLRC4, concomitant with increased generation of the NLRC4 inflammasome activation markers, namely IL-1β and IL-18, as well as TNF-α, in the SN of MPTP-treated mice (Figure 7B,C). Indeed, our finding is in line with previous findings demonstrating GFAP activation in a subacute MPTP mouse model of PD [77,85]. Next, using unbiased stereological analysis, we confirmed that MPTP induced a marked reduction in TH^+^ immunoreactive neurons in the SN of MPTP-treated mice as compared with the saline group (Figure 7D) [77]. Thus, TWEAK expression within activated astrocytes and resultant activation of the NLRC4 inflammasome is likely to contribute to the progression of DAergic neurotoxicity, presumably via a PKCδ-dependent activation mechanism following MPTP intoxication.

### 4.10. TWEAK Enhances NLRC4 Inflammasome Activation and Astrogliosis in the Striata

Given that astrocytes were responsive to TWEAK in vitro, we further investigated whether TWEAK induced astrogliosis in mouse brains that received an intrastriatal injection of TWEAK using a previously demonstrated TWEAK infusion paradigm [45]. Brains were harvested 24 h post-infusion, and the magnitude of GFAP expression, an astrocyte activation marker, and expression of the NLRC4 inflammasome and proinflammatory markers, were assessed by WB and Luminex ELISA analysis, respectively. Interestingly, TWEAK increased the expression of GFAP with a concomitant upregulation of NLRC4 inflammasome expression levels as well as proinflammatory cytokine generation including IL-1β and TNF-α, as compared with PBS-infused mice, suggesting a role for NLRC4 inflammasome activation in the induction of proinflammatory astrocytic activation in TWEAK-treated mice (Figure 7E–G). To further investigate this phenomenon, we conducted immunofluorescence staining for GFAP to assess astrocyte reactivity. Our IHC studies further confirmed an increased density of GFAP^+^ astrocytes in the SN of TWEAK-infused mice when compared to PBS-infused mice (Figure 7H) [45]. Consistent with our aforementioned in vitro findings and other studies showing that brain injections of TWEAK promote astrogliosis [45,87], our studies demonstrate that TWEAK elicits a prominent astroglial activation response that is accompanied by the NLRC4 inflammasome activation mechanism.

## 5. Discussion

Accumulating evidence supports dysregulated glia-related mechanisms in neuroinflammatory disorders, including PD [88,89]. However, the critical contribution of potential inflammatory mediators in mediating neurodegeneration in the context of PD remains poorly understood. Thus, in this study, we focus on TWEAK, which has been linked to PD-like DAergic neuropathology. Our results show that TWEAK elicits astroglial reactivity via activation of STAT3 and the NLRC4 inflammasome, and the associated generation of proinflammatory cytokines via a PKCδ- and mitochondrial oxidative stress-dependent mechanism in U373 human astrocytic cells in culture. Similarly, we observed enhanced activation of PKCδ and STAT3, and the NLRC4 inflammasome in TWEAK-stimulated primary astrocytes, further supporting TWEAK’s role in dysregulating astroglial function. Notably, we found that inhibition of mitochondrial oxidative stress in astrocytes markedly attenuated TWEAK ACM-induced neurotoxicity in LUHMES human DAergic neuronal cells. Furthermore, PKCδ gene KD attenuated the TWEAK-induced proinflammatory signaling response in U373 astrocytes. A similar response was evidenced in mito-apocynin-treated, TWEAK-stimulated U373 cells, suggesting that mitochondria-mediated oxidative stress mechanisms trigger an astrocytic neurotoxic activation state. Notably, we validated our in vitro findings in an MPTP mouse model of PD, as well as in mice that received intrastriatal administration of TWEAK, whereby a positive correlation between astrocytic reactivity and NLRC4 inflammasome activation was evidenced. Our current findings are indeed consistent with a recent report demonstrating the role of the NLRC4 inflammasome in sterile inflammasome activation in astrocytes [72]. Together, our study provides novel insights into the mechanisms regulating the TWEAK-induced astroglial activation response, especially the pivotal contribution of mitochondrial oxidative stress and PKCδ-dependent activation of the STAT3-NLRC4 signaling axis in stimulating DAergic neurodegeneration. Thus, the PKCδ/NLRC4/STAT3 signaling cascade may represent a novel therapeutic avenue for PD.

Accumulating evidence also supports the role of astrocytic dysfunction as an essential pathological intermediate in the nigrostriatal neurodegeneration associated with PD, possibly by enhancing innate immunity within the brain [85,90,91]. For example, a strong correlation between GFAP activation and the loss of nigral DAergic neurons was evidenced in the SN of postmortem PD brains [91]. Moreover, in a recent in vitro study, fibrillar α-synuclein was found to induce a prominent astrocytic activation response and subsequent DAergic neurodegeneration [82]. Indeed, the present study demonstrates that transferring ACM from TWEAK-treated U373 cells to LUHMES human DAergic neuronal cells induced DAergic neurotoxicity. Intriguingly, inhibition of mitochondrial oxidative stress via mito-apocynin suppressed TWEAK-induced DAergic neurotoxicity, suggesting that TWEAK-induced mitochondrial oxidative stress in U373 cells may secrete neurotoxic factors that facilitate DAergic neuronal loss. In line with our findings, a recent study [91] reported that astrocytic KO of NFκB blocks neuroinflammation and oxidative stress, thereby contributing to neuroprotection in an MPTP mouse model of PD. Thus, consistent with previous reports [12,92,93,94], our study suggests that the astrocytic oxidative stress response may contribute to PD pathogenesis, thus providing a possible mechanism linking DAergic neuronal cell death to the astrocytic neurotoxic activation state. 

Oxidative stress has been attributed to an imbalance in the antioxidant/ROS generating machinery, thereby leading to altered homeostasis. The central role of oxidative stress in PD originates from studies conducted in postmortem PD brains demonstrating elevated levels of oxidized proteins, lipids and nucleic acids [95,96,97,98,99,100]. A few of the oxidative species linked to oxidative stress include superoxide, hydrogen peroxide, hydroxyl and nitrosylated radicals, among others. Furthermore, excessive ROS generation may exert deleterious effects on cellular components such as lipids, proteins and DNA, thereby exacerbating oxidative damage [101,102]. ROS generation has been identified as an upstream signaling intermediate leading to mitochondrial dysfunction upon TWEAK stimulation of HUVECs [56]. In this context, ROS has been identified as a key contributor to the collapse of mitochondrial function during TWEAK-induced oxidative damage [61]. In line with these findings, the present study demonstrates that TWEAK treatment induces a significant time-dependent increase in ROS generation with a coincident loss of mitochondrial morphology in TWEAK-stimulated U373 cells. Moreover, it is plausible that TWEAK-induced mitochondrial dysfunction further accentuates the oxidative stress response. This hypothesis is supported by the present study, whereby mito-apocynin inhibited ROS generation while concomitantly preserving mitochondrial function, pointing to astroglial mitochondria as a significant source of ROS in response to TWEAK. Alternatively, mito-apocynin can block the mitochondrial oxidative stress response concomitant with PKCδ activation, leading to the attenuation of the astrocytic neurotoxic activation state and the eventual loss of DAergic neuronal viability. Thus, our study raises the possibility that TWEAK-induced mitochondrial impairment and resultant ROS generation modulates not only the induction of proinflammatory signaling in U373 astrocytes, but also the release of neurotoxic mediators that contribute to the demise of DAergic neurons.

Despite sparse information on the relationship between astrocytic PKCδ activation and PD pathogenesis, previously, we reported the functional coupling between PKCδ activation and PD [39] in microglia, as well as its activation in response to diverse inflammagens, including TNF-α, LPS and aggregated a-synuclein. In our study, TWEAK induced a pronounced increase in PKCδ activation in both U373 astrocytes and primary astrocytes. The siRNA targeted against PKCδ significantly downregulated TWEAK-induced PKCδ activation in U373 cells with an accompanying reduction in the oxidative stress response, suggesting crosstalk between PKCδ and the oxidative stress response in driving the astrocytic activation response. Moreover, siRNA-mediated PKCδ KD attenuated TWEAK-induced NLRC4 inflammasome activation and release of the proinflammatory factors including IL-1β, TNF-α and IL-18. These findings highlight the functional interaction between PKCδ and the oxidative stress response during TWEAK-induced astroglial NLRC4 inflammasome activation, as well as the subsequent release of proinflammatory cytokines. 

STAT3 is a transcription factor that contributes to innate immunity through the release of proinflammatory cytokines [64]. STAT3 homodimerizes, autophosphorylates and subsequently translocates to the nucleus, where it binds to the enhancer regions of the IL-6 promoter, thereby promoting gene transcription [103]. Activation of the JAK/STAT3 pathway has been observed in reactive astrocytes in several conditions of acute injury [104]. Upregulated STAT3 found in reactive astrocytes promotes the metastasis of brain tumors in patients [105]. Moreover, in response to a proinflammatory stimulus, such as LPS or HIV1 trans-activator of transcription (TAT), levels of proinflammatory cytokines, such as IL-6, are elevated via a STAT3 activation mechanism in microglia [64,106]. Additionally, elevated levels of ROS have been reported to be a crucial factor for STAT3 activation [107,108]. Despite these studies, the molecular mechanisms underlying STAT3 activation in astrocytes remain poorly characterized. In this study, we hypothesized that TWEAK-driven mitoROS production induces the activation of STAT3. As expected, TWEAK-induced STAT3 activation and proinflammatory cytokine release were abolished by the mitochondrially-targeted antioxidant, mito-apocynin, indicating that mitochondrial oxidative stress is involved in TWEAK-induced STAT3 activation and the resultant release of proinflammatory cytokines. Intriguingly, a previous report demonstrated that inhibiting PKCδ with a phosphorylation-defective mutant abolished STAT3 phosphorylation at ser 727 during insulin-induced keratinocyte proliferation. Similar to that study, we report here that PKCδ gene silencing decreased STAT3 activation in TWEAK-stimulated astrocytic cells. However, the contribution of PKCδ KD-mediated reduction in oxidative stress to downregulating the STAT3 activation response in TWEAK-treated U373 cells cannot be entirely ruled out. Alternatively, it is also possible that mito-apocynin impacts other anti-inflammatory signaling events to influence the TWEAK-induced astroglial activation response. Future studies will examine whether mito-apocynin regulates other inflammation-related pathways in addition to other transcription factors to modulate the levels of TWEAK-induced proinflammatory cytokine release. 

Recent studies support the concept that inflammaging is a critical contributor to the onset and progression of neurodegenerative diseases. Accordingly, it has been postulated that inflammaging may represent a pivotal mechanism contributing to PD [109]. The inflammasome, which is a component of the innate immune response, participates in the activation of proinflammatory caspase-1 and the resultant proteolytic processing of the proinflammatory cytokines interleukin IL-1β and IL-18. Previous studies have demonstrated that in vitro stimulation of astrocytes with TWEAK leads to the release of IL-6 and IL-8 [38]. Thus, TWEAK treatment of U373 cells may result in the release of proinflammatory cytokines via NLRC4 inflammasome activation. The NLRC4 inflammasome is considered to be a sensor for pathogenic bacteria; however, a growing body of evidence supports its role in sterile injuries, such as ischemic brain injury [110], and its levels were found to be elevated in the brain tissues of AD patients. Furthermore, it is known to participate in palmitate-induced inflammasome activation in astrocytes in culture [28]. Additionally, NLRC4 inflammasome expression is upregulated by proinflammatory stimuli such as TNF-α [29]. To the best of our knowledge, the current work provides the first evidence of NLRC4 inflammasome activation in TWEAK-stimulated astrocytes. NLRC4 expression in glial cells is elevated during the chronic stages of neuroinflammation in the cuprizone model of neuroinflammation and demyelination [72]. Considering that the role of inflammasomes in mediating astrocyte activation is poorly understood, the novelty of this study lies in its deciphering the role of TWEAK-induced NLRC4 inflammasome activation in astrocytes, although the role of NLRP3 inflammasome activation in mediating TWEAK-induced astroglial proinflammatory cytokine release cannot be excluded. In this context, MCC950, an NLRP3 inflammasome inhibitor, was found to mitigate the TWEAK-induced increase in inflammasome activation marker release in U373 astrocytic cells. Furthermore, a growing body of evidence implicates transcriptional and posttranscriptional regulatory mechanisms in the NLRC4 inflammasome activation mechanism [75,111,112,113,114]. In this context, phosphorylation- and ubiquitination-dependent transcriptional regulation are the two best-described regulatory mechanisms in inflammasome activation [115,116]. For example, in a previous report, NLRC4 activation was found to be regulated via PKCδ-dependent phosphorylation of NLRC4, and that PKCδ deficiency greatly attenuated caspase-1 activation and IL-1b secretion in response to the pathogen *Salmonella typhimurium* [74]. In accordance with this finding, NLRC4 inflammasome activation in the present study was preceded by the time-dependent enhanced phosphorylation of PKCδ in TWEAK-stimulated astrocytes and, as anticipated, PKCδ gene depletion via RNAi suppressed NLRC4 inflammasome activation. These data suggest that NLRC4 inflammasome activation may be linked to TWEAK-induced proinflammatory cytokine secretion, including IL-1β, in astrocytes. The importance of NLRC4 inflammasome activation in the CNS is further supported by findings in the MPTP mouse model, whereby increased expression of the NLRC4 inflammasome and its activation markers were upregulated in the SN of these mice. Intriguingly, a positive association between the NLRC4 inflammasome and PKCδ activation, as well as TH neuronal loss, was evidenced in the SN of MPTP-treated mice. While examining MPTP DAergic neurotoxicity, we discovered partial TWEAK colocalization in GFAP^+^ astrocytes as compared to saline-treated mice. Our results contradict a previous finding demonstrating a lack of change in TWEAK expression levels in the SN of MPTP-treated mice [37]. This lack of congruency may be partly attributed to methodological differences and overlooking TWEAK-associated astroglial reactivity in the SN of MPTP-treated mice. To further validate TWEAK-induced dysregulation of astroglial function, we examined astroglial reactivity in mice that received an intrastriatal infusion of TWEAK. As anticipated, TWEAK intrastriatal infusion induced the expression of GFAP protein, which positively correlated with increased NLRC4 inflammasome expression and proinflammatory cytokine levels in the SN. Moreover, morphological analysis of ipsilateral SNpc astrocytes 24 h after TWEAK infusion showed greatly activated astrocytes, as defined by increased GFAP expression relative to controls. Taken together, our data suggest that TWEAK-induced astroglial reactivity may predispose nigral DAergic neurons to PD-like pathology. Additionally, targeting mitochondrial oxidative stress-dependent proinflammatory cell signaling events in astrocytes may facilitate the development of novel therapeutics for slowing or stopping the progression of this insidious disease.

## 6. Conclusions

In summary, this work highlights the critical contribution of PKCδ and the mitochondrial oxidative stress response during TWEAK-induced dysregulation of astroglial function. As schematized (Figure 7I), these results illustrate a novel mechanism by which PKCδ mediates NLRC4 inflammasome and STAT3 activation in astrocytic cells through the regulation of mitochondrial oxidative stress, which, in turn, may partly contribute to the induction of DAergic neuronal loss, possibly via excessive generation of the neurotoxic cytokine IL-1β. Importantly, increased TWEAK expression within reactive astrocytes positively correlated with PKCδ and NLRC4 inflammasome activation, concurrent with TH neuron loss in an MPTP mouse model, suggesting that astrocytic dysfunction may represent a key pathological mechanism contributing to the degeneration of DAergic neurons in PD. Considering the pivotal contribution of astrocytes in neuronal well-being, these findings have broader implications for an improved understanding of the molecular mechanisms that drive astrocytic dysfunction in neuroinflammation-related disorders, including PD.

## Supplementary Materials

The following are available online at https://www.mdpi.com/2073-4409/9/8/1831/s1, Figure S1: Effect of TWEAK on U373 astroglial cell viability, Figure S2: Activation of PKCδ and NLRC4 inflammasome related markers in response to TWEAK stimulation of U373 cells is dose dependent, Figure S3: Downregulation of PKCδ protein expression upon targeted siRNA mediated gene silencing of PKCδ in U373 cells, Figure S4: TWEAK stimulation of U373 astrocytes does not impact PKCδ mRNA expression levels. And Figure S5: Inhibition of NLRP3 inflammasome activation mitigated TWEAK-induced upregulation of proinflammatory cytokine secretion in U373 cells.
