# Supplementary material for: Tumor Necrosis Factor-Like Weak Inducer of Apoptosis (TWEAK) Enhances Activation of STAT3/NLRC4 Inflammasome Signaling Axis through PKCδ in Astrocytes: Implications for Parkinson’s Disease
Source: Cells. 2020 Aug 4;9(8):1831. doi: 10.3390/cells9081831 (PMC7464730; doi:10.3390/cells9081831)
Supplement: Supplementary file 1 [file cells-09-01831-s001.pdf]

Article

Tumor Necrosis Factor-like Weak Inducer of Apoptosis (TWEAK) Enhances Activation of STAT3/NLRC4 Inflammasome Signaling Axis through PKCδ in Astrocytes: Implications for Parkinson's Disease

Manikandan Samidurai [1], Prashant Tarale [1], Chelva Janarthanam [1], Crystal Gomez Estrada [1], Richard Gordon [2], Gary Zenitsky [1], Huajun Jin [1], Vellareddy Anantharam [1], Anumantha G. Kanthasamy [1] and Arthi Kanthasamy [1,*]

1 Department of Biomedical Sciences, Iowa Center for Advanced Neurotoxicology, Iowa State University, Ames, IA 50011, USA; manisk@iastate.edu (M.S.); ptarale@iastate.edu (P.T.); chelvaj@iastate.edu; (C.J.); crystalgomezestrada@gmail.com (C.G.E.); zenitsky@iastate.edu (G.Z.); egb761@iastate.edu (H.J.); anantram@iastate.edu (V.A.); akanthas@iastate.edu (A.G.K.)

2 School of Biomedical Sciences, University of Queensland, St Lucia, Queensland 4072, Australia; r.gordon1@uq.edu.au

* Correspondence: arthik@iastate.edu; Tel.: (515)-294-7238; Fax: (515)-294-2315

Received: 8 July 2020; Accepted: 31 July 2020; Published: date

Supplementary material

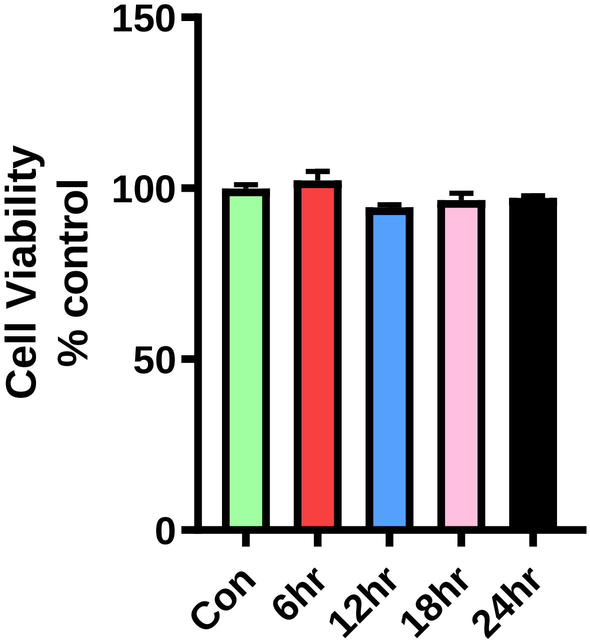

Figure 1. Effect of TWEAK on U373 astroglial cell viability. Human astrocyte (U373) cells were treated with TWEAK (100 ng/mL) for increasing time points (6 h, 12 h, 18 h, and 24 h) and then assayed for the cell viability using MTS cell viability assay. Histograms depict the % of viable cells following TWEAK treatment for the indicated time points. 100

ng/mL TWEAK exposure failed to induce loss of cell viability in U373 human astrocytic cells and were comparable to vehicle treated cells. Data are represented as Mean ± SEM from at least 4 independent experiments.

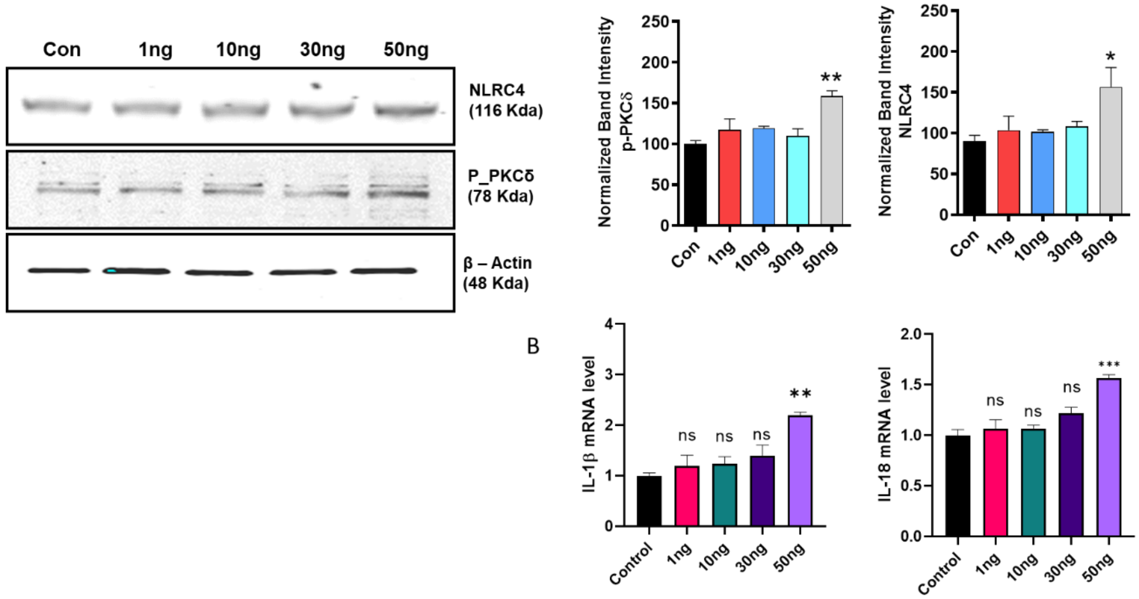

Figure S2. Activation of PKCδ and NLRC4 inflammasome related markers in response to TWEAK stimulation of U373 cells is dose dependent. (**A-B**) Human astrocyte (U373) cells were stimulated with increasing concentrations of TWEAK (1ng, 10ng, 30ng, and 50ng) for 24h and analyzed thereafter for PKCδ phosphorylation, NLRC4 through Western blotting analysis and band quantification performed using densitometric scanning analysis (**A**) Proinflammatory cytokine mRNA expression levels of IL-1β and IL-18 were assessed by qPCR analysis. Data are represented as Mean ± SEM from at least 3 independent experiments. Data were analyzed using the one-way ANOVA followed by Bonferroni's post-Hoc analysis. Asterisks (***$p < 0.001$, **$p < 0.01$ and *$p < 0.05$) indicate significant differences between control and treatment groups.

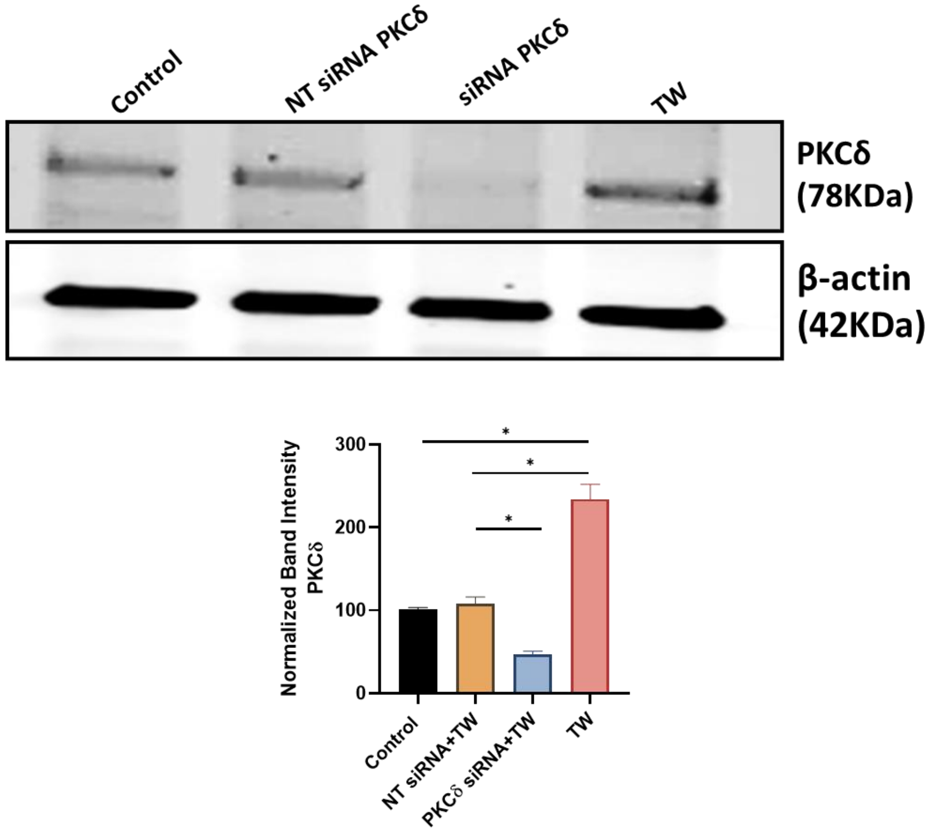

Figure S3. Down regulation of PKCδ protein expression upon targeted siRNA mediated gene silencing of PKCδ in U373 cells. Human astrocyte (U373) cells were transfected with scrambled non target siRNA or PKCδ siRNA and incubated for 48h and subsequently exposed to TWEAK for another 24 h. At the end of the treatment period cell lysates were prepared and the protein expression levels of PKCδ was determined by Western blot analysis. Representative blots and band quantification by densitometric scanning analysis reveal that PKCδ gene knock down resulted in a significant reduction in the endogenous expression levels of PKCδ (70-75%) in U373 cells as compared with scrambled siRNA transfected cells. Data are represented as Mean ± SEM from at least 4 independent experiments. Data were analyzed using the one-way ANOVA followed by Bonferroni's post-Hoc analysis. Asterisks (*$p < 0.05$) indicate significant differences between control and treatment groups.

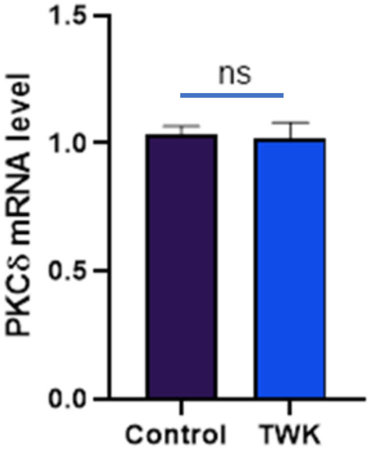

Figure S4. TWEAK stimulation of U373 astrocytes does not impact PKCδ mRNA expression levels. Human astrocyte (U373) cells were treated with TWEAK (100ng) for 24h and analyzed thereafter for PKCδ mRNA expression through qPCR analysis. TWEAK has no significant effect on PKCδ gene expression at 24 h. Data are represented as Mean ± SEM from at least 3 independent experiments. Data were analyzed using the t-test followed by two tailed analysis. (NS): not significant.

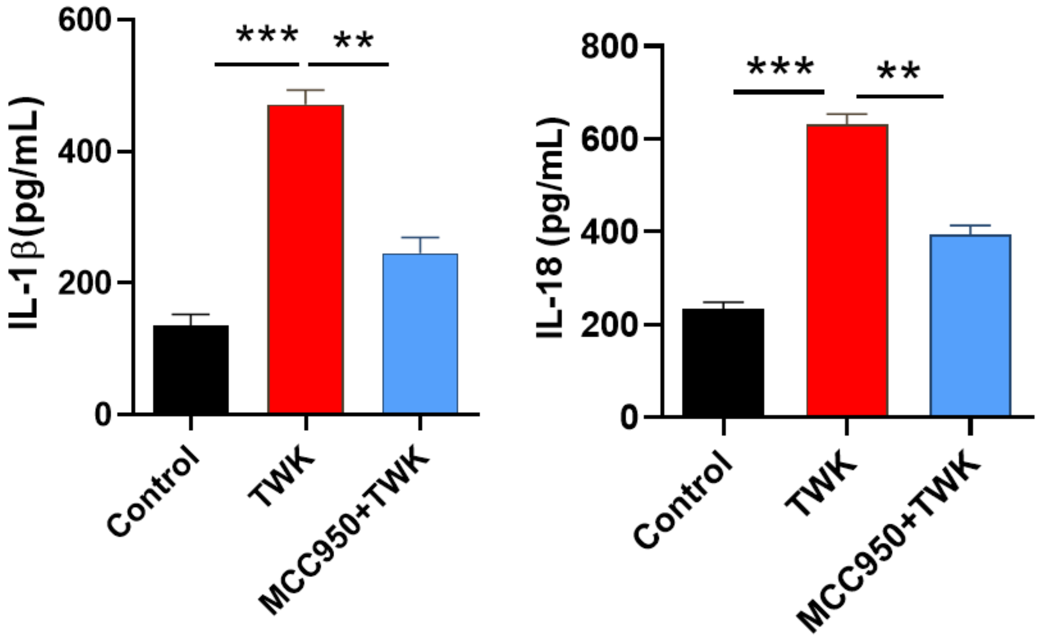

Figure S5. Inhibition of NLRP3 inflammasome activation mitigated TWEAK-induced upregulation of proinflammatory cytokine secretion in U373 cells. Stimulation of U373 cells with TWEAK resulted in increased the release of IL-1β and IL-18 into the extracellular media at 24 h. Pretreatment with MCC950 significantly reduced the secretion of IL-1 β and IL-18 as measured by ELISA analysis. U373 astrocytic cells were stimulated with TWEAK in the presence or absence of MCC 950 and at the end of the incubation period media washarvested and measured for the afore mentioned proinflammatory cytokines using ELISA analysis. Data are represented as Mean ± SEM from at least 3 independent experiments. Data were analyzed using the one-way ANOVA followed by Bonferroni's post-Hoc analysis. Asterisks (***p < 0.001, **p < 0.01) indicate significant differences between control and treatment groups.
